# Induction of IL-22-Producing CD4+ T Cells by Segmented Filamentous Bacteria Independent of Classical Th17 Cells

**DOI:** 10.3389/fimmu.2021.671331

**Published:** 2021-09-08

**Authors:** Urmi Roy, Rômulo S. de Oliveira, Eric J. C. Galvez, Achim Gronow, Marijana Basic, Laura Garcia Perez, Nicola Gagliani, Andre Bleich, Samuel Huber, Till Strowig

**Affiliations:** ^1^Department of Microbial Immune Regulation, Helmholtz Centre for Infection Research, Braunschweig, Germany; ^2^Institute for Laboratory Animal Science, Hannover Medical School, Hannover, Germany; ^3^I. Department of Medicine, University Medical Center Hamburg-Eppendorf, Hamburg, Germany; ^4^Department of General, Visceral and Thoracic Surgery, University Medical Center Hamburg-Eppendorf, Hamburg, Germany

**Keywords:** segmented filamentous bacteria (SFB), cytokine knock-in reporter mice, IL-22, Th22 cells, bystander activation of T cells, *Salmonella* infection

## Abstract

The intestinal microbiota modulates IL-22 production in the intestine, including the induction of IL-22-producing CD4+ T helper cells. Which specific bacteria are responsible for the induction of these cells is less well understood. Here, we demonstrate through the use of novel gnotobiotic knock-in reporter mice that segmented filamentous bacteria (SFB), which are known for their ability to induce Th17 cells, also induce distinct IL-17A negative CD4+ T cell populations in the intestine. A subset of these cells instead produces IL-22 upon restimulation *ex vivo* and also during enteric infections. Furthermore, they produce a distinct set of cytokines compared to Th17 cells including the differential expression of IL-17F and IFN-γ. Importantly, genetic models demonstrate that these cells, presumably Th22 cells, develop independently of intestinal Th17 cells. Together, our data identifies that besides Th17, SFB also induces CD4+ T cell populations, which serve as immediate source of IL-22 during intestinal inflammation.

## Introduction

Antigen-specific CD4+ T cells play pivotal roles during immune responses differentiating into diverse subsets of T helper (Th) cells. These secrete potent immunoregulatory cytokines and form long-lasting memory. Th17 and Th22 are two subsets of CD4+ T cells that are considered crucial for maintaining the balance between homeostasis and inflammation at mucosal sites. Th17 cells are characterized by their production of effector cytokines interleukin (IL)-17A, IL-17F, and IL-22, thereby functioning as important activators of innate immune effectors and contributing to the mucosal defense against extracellular bacteria and fungi (1, 2). Pathogenic roles of Th17 cells have also been described in different animal models of autoimmune disease including encephalomyelitis (3–5), rheumatoid arthritis (6, 7), psoriasis (8, 9), multiple sclerosis (10, 11), and inflammatory bowel disease (12–14). In humans, Th22 cells are distinguished from Th17 cells based on the lack of IL-17A expression and are considered the major source of adaptive immune cell-derived IL-22 (15, 16). As the effector molecule IL-22 confers both proinflammatory and/or tissue protective functions based on the type of inflammatory response, cellular source, and surrounding cytokine milieu (17, 18).

Studies performed in different *in vivo* and *in vitro* models have shown a high heterogeneity of both Th17 and Th22 cell subsets characterized by the variable production of additional immunomodulatory cytokines such as IFN-γ and IL-10 (19, 20). Notably, multi-parameter flow cytometry has identified the presence of numerous tissue-specific Th cell subsets with distinct cytokine expression throughout the intestine and other tissues in the human body (21). Yet, detailed studies are still needed to further identify the instructive networks as well as both the molecular and functional properties of potentially heterogenous Th17 and Th22 cell subsets.

The making of lineage-decisions during T cell differentiation is heavily influenced by environmental stimuli. In the gastrointestinal (GI) tract, hundreds of distinct species of microorganisms constantly convey signals to the host mucosal immune system. However, it is evident now that only specific members of the intestinal microbiota are able to induce antigen-specific helper CD4+ T cells in the absence of a classical infection. The eminent example are the segmented filamentous bacteria (SFB), which induce the differentiation of Th17 cells (22, 23). A subsequent study suggested that intestinal Th17 cells exclusively harbor SFB specific T cell receptor (TCR) responding upon exposure to SFB antigen (24). Notably, besides SFB, distinct pathogens and other human-derived intestinal bacteria with the ability to attach to the epithelium as well as fungi have been demonstrated to induce Th17 cell differentiation (25).

Besides the classical antigen-dependent activation of T cells, which is observed for Th17 cells in the small intestine where SFB resides, bystander activation, i.e., the stimulation of memory T cells by cytokines during an immune response or inflammation, has emerged as a potent activation mechanism of tissue-resident memory cells (26, 27). Specifically, the protection mediated by SFB-induced Th17 cells during *C. rodentium* infection has been hypothesized to occur upon bystander activation (22). Along these lines, we were able to show that distinct members of the microbiota induced the enhancement of IFN-γ production by CD4+ T cells, which contributed to the resistance of mice against *Salmonella* infection (28). While these results provide more evidence for the impact of the microbiota on CD4+ T cells in the intestine, it is not known yet, whether CD4+ T cells and specifically Th17 and Th22 cells primed by the intestinal microbiota produce the same effector cytokines during antigen-induced and bystander activation *in vivo*.

Importantly, Th17 and Th22 cells have been characterized in detail *in vitro*, however, technical difficulties such as the inability to determine gene expression profiles after *ex vivo* restimulation and fixation of the cells have precluded a detailed *ex vivo* characterization of effector functions and diversity *in vivo*.

## Methods

### Mice

Wild type (WT) and all transgenic lines, IL-17A^GFP^ IL-22^BFP^ FoxP3^RFP^, RORγt^GFP^ FoxP3^RFP^, IL-17A^GFP^ IFN-γ^Katushka^ FoxP3^RFP^, IL-17A^GFP^ IL-22^BFP^ IFN-γ^Katushka^ FoxP3^RFP^, IL-17A^CRE^ R26^YFP^, CD4dnTGFβRII, and *Rag2^-/-^* mice used in the study were on the C57BL/6N background and have been rederived into an enhanced specific pathogen free (eSPF) microbiota by embryo transfer and bred at the specific pathogen free animal facilities of Helmholtz Centre for Infection Research (HZI). SFB-monocolonized NOD.CB17-*Prkdc* ^scid^/J mice were bred in gnotobiotic isolators at the Medical University Hannover, Germany. Colonization with SFB was achieved by cohousing or oral gavage (protocol described below) with the intestinal content from SFB-monocolonized mice at least four weeks before experiments. Germ-free C57BL/6NTac and IL-17A^GFP^ IL-22^BFP^ FoxP3^RFP^ mice were bred in the isolators (Getinge) in the germ free facility of the HZI. All experiments were performed with 8–12 weeks old age-matched and gender-matched animals. Both male and female animals were used for every experiment to exclude the influence of gender.

### Fecal Transplantation of Contents From Segmented Filamentous Bacteria Monocolonized Mice

For fecal transplantation experiments, donor mice were euthanized, intestinal content was collected from large intestine, cecum, and SI and pre-homogenized in BBL thioglycollate media (BD Bioscience) by vortexing. Under anaerobic conditions, fecal content was homogenized using a 70-μm sterile filter. After centrifugation (10 min, 500 g, 4°C), fecal material was resuspended in BHI medium (Sigma-Aldrich). A total of 200 μL of fecal transplant was given by oral gavage to recipient mice, which were starved 2 h prior. Four weeks after cohousing or fecal transplant gastrointestinal colitis was induced by infection with *S. typhimurium*.

### Colitis Model Induced by Infection With *S. typhimurium*

Naturally streptomycin-resistant wild-type strain *S. enterica* serovar Typhimurium SL1344 was used. *Salmonella* strains were grown overnight at 37°C in Luria-Bertani (LB) broth, with 50 µg/ml kanamycin then, diluted 1:100 in a fresh medium, and subcultured for 4 h. Bacteria were washed twice in an ice-cold phosphate-buffered saline (PBS) and then used for infection experiments. Water and food were withdrawn for 4 h before mice were treated with 20 mg/mouse of streptomycin by oral gavage (o.g.). Afterwards, mice were supplied with water and food *ad libitum*. Then, 20 h after streptomycin treatment, water and food were withdrawn again 4 h before the mice were infected with 10^5^ CFU of *S. typhimurium* in 200 µl of PBS. Drinking water *ad libitum* was supplied immediately and food 2 h post infection (p.i.). Mice were weighted every day and survival was monitored.

### Analysis of Segmented Filamentous Bacteria Loads From Content and Tissue

Fresh content samples of mice were collected from the small intestine and colon and immediately stored at -20°C. DNA was extracted according to the established protocols using a method combining mechanical disruption (bead-beating) and phenol/chloroform-based purification (29). Briefly, the sample was suspended in a solution containing 500 µl of extraction buffer (200 mM Tris, 20 mM EDTA, 200 mM NaCl, pH 8.0), 200 µl of 20% SDS, 500 µl of phenol:chloroform:isoamyl alcohol (24:24:1), and 100 µl of 0.1 mm zirconia/silica. Samples were homogenized twice with a bead beater (BioSpec) for 2 min. After the precipitation of DNA, crude DNA extracts were resuspended in TE Buffer with 100 µg/ml RNase and column purified to remove PCR inhibitors (BioBasic).

To monitor the tissue association of SFB, tissue samples were collected from the small intestine, cecum, and colon. Briefly, intestinal tissues were opened longitudinally, washed with autoclaved PBS to clean, and homogenized in TriReagent (Molecular Research Center) using mechanical disruption (bead-beating). RNA was isolated according to the instructions of the manufacturer and treated with 2 U of DNAase I (Ambion) for 25 min at 37°C. One microgram of total RNA was used to generate cDNA (RevertAid Reverse Transcriptase) using random hexamer primers.

Quantitative PCR of isolated DNA samples from intestinal content and cDNA from intestinal tissue samples was performed to detect total bacteria and SFB using specific primer sets: 16S (F: 5’- ACTCCTACGGGAGGCAGCAGT and R: 5’- ATTACCGCGGCTGCTGGC) and SFB (F: 5’- GACGCTGAGGCATGAGAGCAT and R: 5’- GACGGCACGGATTGTTATTCA).

### Field Emission Scanning Electron Microscopy

Gut samples were fixed with 5% formaldehyde and 2% glutaraldehyde in HEPES buffer (0.1 M of HEPES, 10 mM of CaCl2, 10 mM of MgCl2, and 0.09 M of sucrose, pH 6.9) overnight at 7°C, then washed twice with TE buffer (20 mM of TRIS, 2 mM of EDTA, pH 6.9), and the gut content was squeezed out. Dehydration was achieved with a graded series of acetone (10%, 30%, 50%, 70%, 90%, 100%) on ice for 15 min for each step. Samples in the 100% acetone step were allowed to reach room temperature before another change in 100% acetone. Samples were then subjected to a critical point drying with liquid CO_2_ (CPD 300, Leica, Wetzlar). Dried samples were covered with a gold-palladium film by sputter coating (SCD 500 Bal-Tec, Liechtenstein) before examination in a field emission scanning electron microscope Zeiss Merlin (Oberkochen) using the Everhart-Thornley SE-detector and the Inlens SE-detector in a 40:60 ratio with an acceleration voltage of 5 kV.

### Isolation of Lamina Propria Leukocytes and Flow Cytometry

To isolate LPL, density gradient centrifugation using Percoll was done as previously described (30). In brief, intestinal organs (small intestine and cecum) were collected during a steady state and 20 h post *S*. *tm.* infection. Fecal content was removed, tissues were opened longitudinally, washed with PBS, and then shaken in HBSS containing 2 mM of EDTA for 20 min at 37°C. Tissues were cut into small pieces and incubated with a digestion solution (DMEM containing 1% fetal bovine serum (FBS), 0.25 mg/ml collagenase D, 0.5 U/ml dispase, and 5 μg/ml DNase I) in a shaker for 20 min at 37°C. Digested tissues were filtered through a 70-µM cell strainer (Falcon) and DMEM + 5% FBS was added to inactivate enzymes. The last two steps were repeated until all tissue was digested. After centrifugation, cells were resuspended in 4 ml of 40% Percoll (GE Healthcare) and overlaid on 4 ml of 80% Percoll. Percoll gradient separation was performed by centrifugation at 450 g for 25 min at 25°C. Cells in the interphase were collected and used as LPL. The collected cells were then suspended in a staining buffer containing PBS, 1% FBS, and 2 mM of EDTA. The following antibodies were used: anti-CD45 (30-F11), anti-CD3 (17A2), anti-CD4 (RM4-5, GK1.5), anti-CD44 (IM7), anti-CD62L (MEL-14), anti-CCR6 (29-2L17) (Biolegend), and anti-Vβ-14(14-2) (BD Bioscience). To distinguish live from dead cells, AlexaFluor-350 NHS Ester (Life Technologies) was used. Flow cytometry analysis was performed using a BD LSR (BD Biosciences) and data were analyzed with FlowJo software (TreeStar Inc.).

To monitor the cytokine production by intestinal LPL during a steady state, isolated LPL cells were cultured in an enriched RPMI-1640 media containing 10% FBS, antibiotic cocktail (100 U/ml penicillin, 100 ug/ml streptomycin, GIBCO, Life technologies), and 2 mM/ml L-Glutamine (GIBCO, Life technologies) for 5 h at 37°C. *Ex vivo* stimulations were carried out in the presence of 50 ng/ml phorbol 12-myristate 13-acetate (PMA) (Sigma), 1 ug/ml Ionomycin (Sigma), 50 ng/ml mouse IL-6 (Biolegend), and 240 ng/ml mouse IL-23 (Biolegend). For the detection of intracecllular cytokines or transcription factors in non-reporter WT mice, stimulated LPL cells were fixed and permeabilized using the Fixation and Permeabilization buffer sets (Biolegend) according to the instructions of the manufacturer or the Foxp3/transcription factor staining buffer set (eBioscience), respectively. Antibodies used for staining were anti-IFNγ (XMG1.2, Biolegend), anti-IL-17A (TC11-18H10.1, Biolegend), anti-IL-22 (1H8PWSR, eBioscience), anti-AhR (T49-550, BD Bioscience), and anti-Rorγt (Q31-378, BD Bioscience).

### Low-Input RNA Sequencing and Quantitative PCR

A minimum of 1,000 cells was sorted, and RNA was isolated using the RNAeasy plus micro Kit (Qiagen). Total RNA was quantified and its quality was assessed using a 2100 Bioanalyzer Instrument (Agilent Technologies). RNA samples with RNA integrity number values >9 were used for cDNA synthesis. A total of 200 pg of isolated RNA from each sample were used to synthesize and amplify cDNA (SMART-Seq v4 Ultra low input RNA Kit, Takara) followed by DNA library preparation for sequencing (Nextera XT, Illumina). This approach allowed us to multiplex up to 12 samples/HiSeq run. We employed an analysis pipeline to determine the differentially expressed genes. Briefly, raw reads were quality filtered using Trimmomatic and aligned to the mouse reference genome (mm10) using STAR. Normalization and differential expression were quantified using the DEseq2 package. Differential expressed gene networks were analyzed using the Consensus Path DB-mouse webserver.

Additionally, quantitative-PCR was performed from the low-input cDNA samples using gene-specific primer sets (Sigma) of *Il17a* primer (F: 5’- ACGTTTCTCAGCAAACTTAC and R: 5’- CCCCTTTACACCTTCTTTTC); *Il17f* primer (F: 5’- ATACCCAGGAAGACATACTTAG and R: 5’- AGTCCCAACATCAACAGTAG); primer sets (Life Technologies) *Ifng* primer (Mm00801778_m1); *Il22* primer (Mm04203745_mH); and Kapa Sybr Fast or Probe qPCR kit (Kapa Biosystems) on a LightCycler 480 instrument (Roche). PCR conditions were 95°C for 60 s, followed by 40 cycles of 95°C for 3 s, and 60°C for 30 s. Data were analyzed using the the deltaCt method with *hprt* (F: CTGGTGAAAAGGACCTCTCG and R: TGAAGTACTCATTATAGTCAAGGGCA) serving as the reference housekeeping gene.

### CD4+ T Cell Co-Transfer Experiment

Total CD4+ T cells were isolated from the spleen and lymph nodes of wild type (CD45.1, CD45.2) and CD4dnTGFbRII (CD45.2) using MACS technology. Further purification was performed using flow cytometry-based cell sorting. A total of two million CD4+ T cells in a 1:1 ratio from both wild type and transgenic mice were adoptively transferred to *Rag2^−/−^* mice through intraperitoneal injection harboring the eSPF+SFB microbiota.

### Statistical Analyses

Statistical analysis was performed using the GraphPad Prism program (GraphPad Software). Data are expressed as mean ± SEM. Differences were analyzed by Student’s t test and ANOVA. *P*-values indicated represent a non-parametric Mann-Whitney U test or Kruskal-Wallis test comparison between groups. *P*-values ≤ 0.05 were considered as significant: *p < 0.05, **p < 0.01, ***p < 0.001, ****p < 0.000.

## Results

### CD4+ T Cells are a Significant Source of IL-17A and IL-22 Production *In Vivo* Upon Enteric Infection

**Figure 1:**
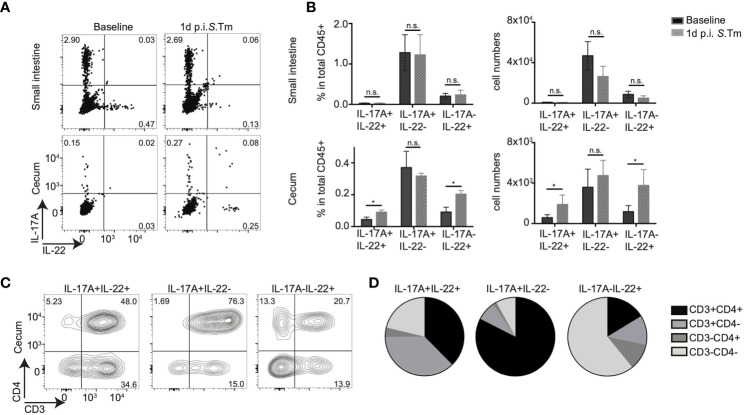
CD4+ T cells contribute to mucosal IL-17A and IL-22 secretion upon bacterial infection. **(A–D)** IL-17A^GFP^ IL-22^BFP^ Foxp3^RFP^ (conventionally raised) mice were infected orally with 10^5^ CFU of *Salmonella enteria* serovar Typhimurium (*S*. *Tm.*) after streptomycin-pretreatment. Lamina propria leukocytes (LPL) were isolated from the small intestine and cecum of uninfected (baseline) and 1 day post *S*. Tm. infected mice. Isolated LPL were analyzed by FACS without any *ex vivo* restimulation. **(A)** Representative FACS plots show IL-17A and IL-22 frequencies gated on total CD45+ cells. In **(B)** frequencies within CD45+, LPL and total numbers of cytokine-producing cells are shown. **(C)** Representative FACS plots analyzing CD3 and CD4 expression gated on IL-17A and/or IL-22 producing cells and relative frequencies of indicated cell subsets within CD45+ cells **(D)**. Data represent n = 4–10 mice/group as mean ± SEM from at least two independent experiments. P-values indicated represent an unpaired Student’s t test *p < 0,05; n.s., not significant. See also **Supplementary Figure S1**.

The cytokines IL-17A and IL-22 play crucial roles during homeostasis and inflammation and are produced by various cell types during mucosal inflammation. To assess the *in situ* production of IL-17A and IL-22, we used a recently described mouse line, the IL-22^BFP^ mice, generated by our collaborators (31), intercrossed with IL-17A^GFP^ and FoxP3^RFP^ mice to generate IL-17A^GFP^ IL-22^BFP^ Foxp3^RFP^ triple reporter mice. Using these mice, we investigated the expression and cellular sources of IL-17A and IL-22 during steady state conditions *in vivo* as well as at an early time point after enteric infections, i.e., *Salmonella typhimurium* infection. Specifically, leukocytes from the intestinal lamina propria (LPL) and lymphoid organs were isolated from conventionally housed IL-17A^GFP^ IL-22^BFP^ Foxp3^RFP^ mice left uninfected (baseline) and 1 day post infection (p. i.) with *S. typhimurium* (*S. tm.*) (**Supplementary Figure S1A**; gating strategy **Supplementary Figure S1B**). Importantly, at this early time point, *S. tm.* has induced significant inflammation in the cecum but not the SI (32) and CD4+ T cells are unlikely to be *S. tm.*-specific but rather activated as bystanders (33). LPLs were not restimulated to reflect *in vivo* cytokine production. We observed that from the total CD45^+^ isolated leukocytes from the small intestine; both the frequencies and absolute numbers of IL-17A and IL-22 cytokine producing cells did not alter upon *S*. *tm.* infection (**Figures 1A, B**). In contrast, among the total cecal leukocyte population, IL-17A^+^IL-22^+^ and IL-17A^-^IL-22^+^ cells were significantly increased after *S. tm.* infection (**Figures 1A, B**). Similar to the small intestine, frequencies and absolute numbers of IL-17A^+^IL-22^-^ cells in the cecum were not affected due to enteric infection (**Figures 1A, B**).

Next, we quantified the relative contribution of CD4+ T cells compared to other immune cell subsets to the production of these cytokines *in situ* within the LPL, i.e., without *ex vivo* restimulation. *In vivo* IL-17A production during a steady state in small intestinal and cecal LPLs was mainly from CD3+CD4+ T cells, likely reflecting the presence of SFB and potentially other IL-17A-inducing bacteria in our conventional mouse facility (data not shown). As expected, since *S*. *tm* induces inflammation mainly in the cecum *in vivo*, IL-17A production from CD3+CD4+ small intestinal LPLs was not altered after infection with *S. tm.* (**Supplementary Figures S1C, D**). In contrast, direct *in situ* IL-22 production was detected almost exclusively in cecal CD45+ LPLs in infected mice, but nearly absent under steady state conditions. Strikingly, CD3+CD4+ cells were the major *in vivo* source of IL-17A single and IL-17A/IL-22 double cytokine production in LPL from an infected cecum (**Figures 1C, D**). Although CD3-CD4- cells were the main source of IL-22 single cytokine production in cecum, CD3+CD4+ cells contributed nearly 20% of IL-22 production after *Salmonella* infection (**Figures 1C, D**). Consequently, a significant increase in IL-17A+IL-22+ and IL-17A-IL-22+ CD4+ T cells was observed upon *S*. *tm.* infection (**Supplementary Figures S1C, D**). Lymphocytes subsets isolated from the mesenteric lymphnodes (mLN) demonstrated no *in vivo* IL-22 production in the absence of restimulation (**Supplementary Figure S1C**). Only IL-17A production was observed from the CD3+CD4+ cell subsets; however, no major difference was detected between the infected and non-infected groups (**Supplementary Figure S1C**). Together, these data suggest that, CD4+ T cells do not express IL-22 in steady state conditions in the intestine and that CD4+ T cells are a source of rapid or “innate-like” IL-22 responses in the cecum mucosa early after *Salmonella* infection.

### *In Vivo* Early IL-17A/IL-22 Response by CD4+ T Cells Upon *Salmonella* Infection Are Dependent on Segmented Filamentous Bacteria

**Figure 2:**
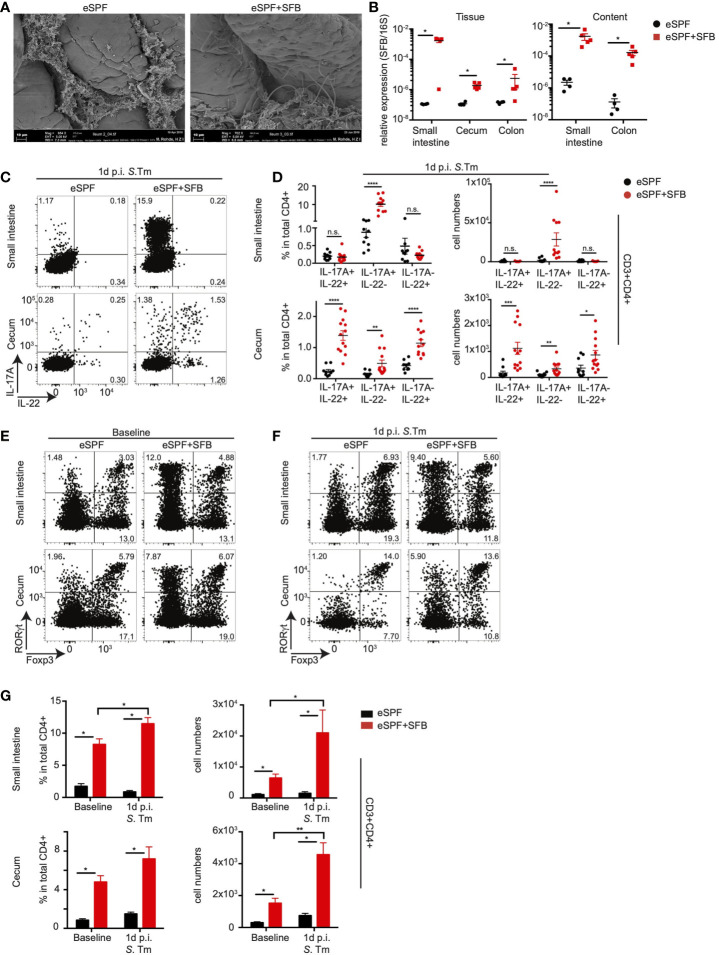
Anti-bacterial IL-17A and IL-22 responses by CD4+ T cells are dependent on SFB. **(A)** Scanning electron microscopy of ileum samples from eSPF mice with or without cohousing with SFB-monocolonized mice. **(B)** Relative expression of SFB specific 16S rRNA gene compared to total bacterial 16S rRNA gene in tissues and contents from different parts of intestinal tract in eSPF and eSPF+SFB colonized mice. **(C, D)** LPL were isolated 1d post *S*. *tm*. infection from the small intestine and cecum of IL-17A^GFP^ IL-22^BFP^ FoxP3^RFP^ mice harboring eSPF or eSPF+SFB. Representative FACS plots and percentages and absolute numbers of CD3+CD4+ cells expressing IL-17A and IL-22 without restimulation. **(E–G)** LPL were isolated during steady state and 1d post *S*. *tm.* infection from small intestine and cecum of RORγt^GFP^ FoxP3^RFP^ mice harboring eSPF or eSPF+SFB. Representative FACS plots **(E, F)** and percentages and absolute numbers **(G)** of CD3+CD4+ cells expressing RORγt and Foxp3. Data represent n = 5–13 mice/group as mean ± SEM from at least two independent experiments. P-values indicated represent an unpaired Student’s t test *p < 0,05; **p < 0,01; ***p < 0,001; ****p < 0,0001; n.s., not significant. See also **Supplementary Figure S2**.

Previous work has demonstrated that “innate-like” Th17 responses upon enteropathogenic infection are dependent on the presence of the intestinal microbiota (26). To further investigate the effect of specific members of the microbiota on *in vivo* cytokine production, we rederived IL-17A^GFP^ IL-22^BFP^ Foxp3^RFP^ mice into an enhanced specific pathogen free (eSPF) microbiota condition lacking numerous known immunomodulatory components of the microbiota, i.e., SFB, *Helicobacter* spp., norovirus, and *Tritrichomonas* spp. that have been reported to influence the quality and quantity of CD4+ T cell responses (22). To determine the influence of SFB on the rapid CD4+ T cell response during Salmonella infection, specifically IL-22 production, we cohoused eSPF reporter mice with SFB monocolonized donor mice for 14 days (**Supplementary Figure S2A**). SFB colonization was confirmed by a scanning electron microscopy of the terminal ileum of SFB cohoused recipients (**Figure 2A**), as well as by performing SFB-specific qPCR of both intestinal mucosa and content (**Figure 2B**). Characterization of IL-17A- and IL-22-producing CD4+ T cells from eSPF and eSPF+SFB reporter mice during steady state conditions and in the absence of restimulation revealed, in line with previous studies, that SFB colonization strongly increased the frequency and number of IL-17A+ CD4+ T cells in the SI (**Supplementary Figures S2B, C**). Notably, while low levels of IL-17 production were detected within CD4+ T cell in the cecum, IL-22 production from CD4+ T cells was undetectable in both the cecum and SI in the absence of restimulation (**Supplementary Figures S2B, C**). Strikingly, infection with *S. tm.* induced in eSPF+SFB mice compared to SPF mice significantly increases the frequencies and numbers of IL-17A-IL-22+ and IL-17A+IL-22+ CD4+ T cells specifically in the cecal LPLs (**Figures 2C, D**). This was similar to the conventionally housed mice suggesting that SFB induces beyond classical Th17 cells also a CD4+ T cell subset that rapid produce in the cecum exclusively IL-22 but not IL-17A after enteric infections.

Prior studies have demonstrated that SFB-induced RORγt+ T cells colonize not only the small intestine but also other mucosal sites (34). Hence, we next quantified the effect of SFB colonization as well as infection on the numbers of cecal tissue-resident RORγt+ T cells in our model. Therefore, we rederived a previously described RORγt^GFP^FoxP3^RFP^ reporter mice (35) into eSPF conditions. This mouse line simplifies the unbiased *in situ* quantification of RORγt- and FoxP3-expressing cell subsets including Th17 cells as well as thymus-derived and periphally induced Tregs. Colonization of eSPF RORγt^GFP^FoxP3^RFP^ reporter mice with SFB resulted in the expansion of RORγt+FoxP3- CD4+ T cells in both the SI and cecum as expected (**Figures 2E, G**). Notably, while *Salmonella* infection did not largely affect the frequencies of RORγt+FoxP3- CD4 T cells in the cecum, their absolute abundance was 2.5-fold higher in eSPF+SFB after infection compared to steady state conditions (**Figures 2F, G**). This suggests that enteric infection already at this early time point results in either *in situ* proliferation or in the recruitment of additional RORγt+FoxP3- CD4+ T cells to the inflamed site. Overall, these results provide support for the previous observation that SFB colonization results in the seeding of RORγt+Foxp3- CD4+ T cells beyond the terminal ileum. Besides, they identifiy that SFB induces, in the context of the eSPF microbiota, the acquisition of diverse cytokine-production profiles in CD4+ T cells, including a subset that produces IL-22 but not IL17A after *Salmonella* infection.

### Segmented Filamentous Bacteria are Sufficient to Induce Distinct IL-17 and IL-22 Responses of CD4 T Cells

**Figure 3:**
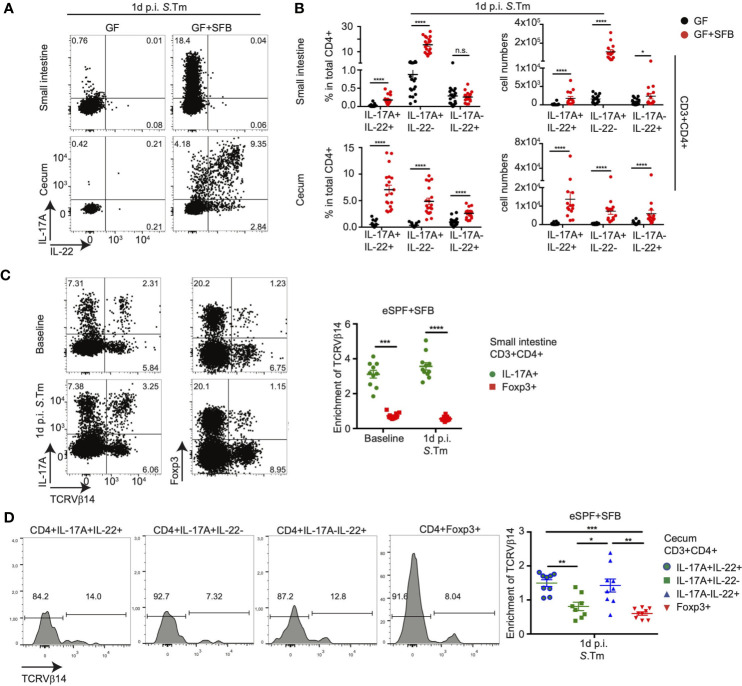
Anti-bacterial IL-17A and IL-22 responses by CD4+ T cells are SFB specific. **(A, B)** LPL were isolated 1d post *S*. *tm.* infection from the small intestine and cecum of IL-17A^GFP^ IL-22^BFP^ FoxP3^RFP^ mice under GF or GF+SFB conditions. Representative FACS plots **(A)** and percentages and absolute numbers **(B)** of CD3+CD4+ cells expressing IL-17A and IL-22 without *ex vivo* restimulation. **(C)** CD3+CD4+ T cells from small intestinal LPL from eSPF+SFB mice were analyzed for Vβ14 expression in IL-17A+ and Foxp3+ cells. Left, representative FACS plots and right, specific enrichment of Vβ14 TCRs in CD4 T cells expressing IL-17A and Foxp3 in the absence of *ex vivo* restimulation. **(D)** CD3+CD4+ T cells from cecal LPL from eSPF+SFB mice after 1d *S*. *tm*. infection were analyzed for Vβ14 expression in IL-17A+ and/or IL-22+ and Foxp3+ cells. Left, representative FACS plots and right, specific enrichment of Vβ14 TCRs in CD4 T cells expressing IL-17A and/or IL-22 and Foxp3 in the absence of *ex vivo* restimulation. Data represent n = 9–16 mice/group as mean ± SEM from at least two independent experiments. P-values indicated represent an unpaired Student’s t test *p < 0,05; **p < 0,01; ***p < 0,001; ****p < 0,0001; n.s., not significant. See also **Supplementary Figure S3**.

The interaction of SFB with other members of the intestinal microbiota has been reported to modulate Th17 functions, for instance, the properties of high salt intake-induced pathogenic Th17 cells (36). To assess whether SFB alone is sufficient to induce the differentiation of distinct subsets of innate-like CD4+ T cells, we rederived IL-17A^GFP^ IL-22^BFP^ Foxp3^RFP^ mice into germ-free (GF) conditions and cohoused them with SFB-monoassociated mice. Similar to SFB cohoused eSPF mice, monoassociation of GF mice with SFB also resulted in a significant production of IL-17A from small intestinal and cecal CD3+CD4+ LPLs at baseline in the absence of *ex vivo* restimulation, notably, even at a higher relative abundance than in the eSPF setting (**Supplementary Figures S3A, B**). During infection with *S*. *tm.*, in line with the observations from the eSPF mice, we detected increased frequencies and absolute numbers of IL-17A-IL-22+ and IL-17A+IL-22+ CD4+ T cells in cecal LPLs from *S. tm.* infected SFB colonized GF mice (**Figures 3A, B**). Unlike eSPF mice, *S. tm.* infection also resulted in a robust accumulation of additional IL-17A+IL-22- CD3+CD4+ T cells in cecal LPL and IL-17A-IL-22+ and IL-17A+IL-22+ CD3+CD4+ T cells also in the small intestinal LPLs of GF mice harboring SFB (**Figures 3A, B**). CD3+CD4- and CD3-CD4- cells did not show any difference in *in vivo* cytokine production in the presence or absence of SFB in GF mice (data not shown). Taken together, these data demonstrate that presence of SFB alone is sufficient for the rapid production of IL-17A and IL-22 by CD4+ T cells after *Salmonella* infection. But, these experiments also highlight the distinct effects of SFB on CD4+ T cells in GF and eSPF mice suggesting that the immunomodulatory effect of SFB is modulated by other commensal bacteria.

It has been demonstrated that SFB-induced Th17 cells present in the SI during steady state conditions are predominantly recognizing distinct epitopes from SFB resulting in the enrichment of T cells expressing the Vβ14 chain of the TCR (24). Hence, we next addressed whether subsets differing in their cytokine-secretion show similar patterns of Vβ14 TCR enrichment after *S. tm.* infection. In line with the previous report, small intestinal IL-17A+ (Th17) cells in steady state conditions were significantly enriched in the Vβ14 TCRs compared to Foxp3+ CD4+ T cells (**Figure 3C**). In cecal LPLs from eSPF+SFB mice infected with *S. tm.*, a significant increase in the Vβ14 TCR enrichment was observed specifically in IL-17A-IL-22+ and IL-17A+IL-22+ CD4+ T cells compared to IL-17A+IL-22- and Foxp3+ CD4+ T cells (**Figure 3D**). We performed similar experiments in SFB monocolonized GF mice and could show the same increase in Vβ14 TCR enrichment in IL-17A-IL-22+ and IL-17A+IL-22+ CD4+ T cell subsets similar to that observed in eSPF+SFB mice (**Supplementary Figures S3C, D**). Unlike in eSPF+SFB mice, increased Vβ14 TCR enrichment was also observed in the IL-17A+IL-22- CD4+ T cell subset in cecal LPLs of GF mice after *S. tm.* infection implying a potentially different antigen specificity within this subset depending on the presence of additional gut microbes (**Supplementary Figure S3D**). Together, these data demonstrate that SFB is sufficient to induce distinct populations of CD4+ T cells characterized by different combinations of innate-like *in situ* IL-17 and IL-22 production upon enteric infection.

### Bystander Activation of Segmented Filamentous Bacteria-Dependent IL-17A and/or IL-22+ CD4+ T Cells has Distinct Gene-Expression Profiles

**Figure 4:**
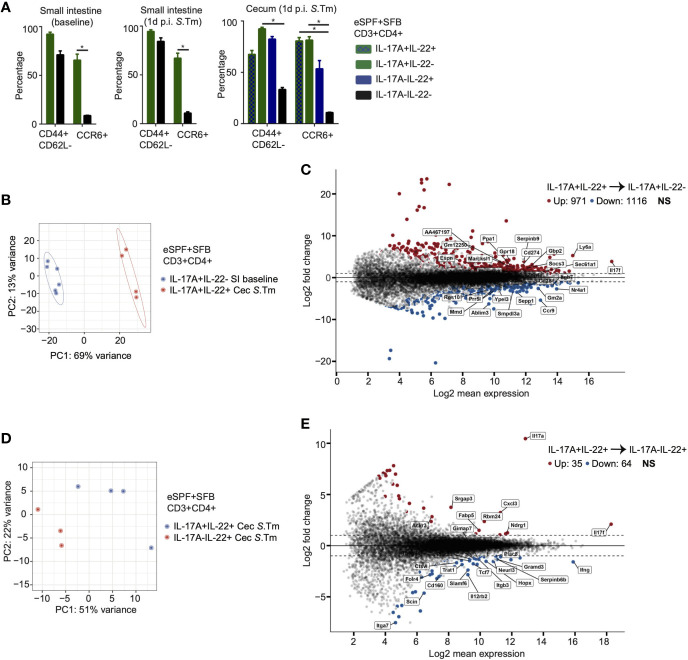
Distinct properties of SFB modulated CD4 T cells. **(A)** CD3+CD4+ T cells from small intestinal and cecal LPL from eSPF+SFB mice during baseline and after 1d *S*. *tm*. infection were analyzed for the expression of CD44, CD62L, and CCR6 expression in IL-17A+ and/or IL-22+ and IL-17A-IL-22- cells in the absence of *ex vivo* restimulation. **(B, C)** RNA-seq analysis of sorted CD3+CD4+ IL-17A+IL-22- cells from small intestinal LPL during baseline and IL-17A+IL-22+ cells from cecal LPL 1d post *S*. *tm*. infection from mice colonized with eSPF+SFB microbiota. β-diversity analysis **(B)** and DEseq analysis **(C)** comparing significant up/downregulation of genes (fold change > 2) of these two subpopulation. Cells were isolated based on cytokine expression in the absence of *ex vivo* restimulation. **(D, E)** RNA-seq analysis of sorted CD3+CD4+ IL-17A+IL-22+ cells and IL-17A-IL-22+ cells from cecal LPL 1d post *S*. *tm*. infection from mice colonized with eSPF+SFB microbiota. β-diversity analysis **(D)** and DEseq analysis **(E)** comparing significant up/downregulation of genes (fold change > 2) of these two subpopulation. Cells were isolated based on cytokine expression in the absence of *ex vivo* restimulation. Data represent n = 4–6 mice/group as mean ± SEM from at least two independent experiments. P-values indicated represent an unpaired Student’s t test *p < 0,05. See also **Supplementary Figure S4**.

Innate-like Th17 cells have been reported to display activated memory-like phenotypes (26, 27). We characterized the activation and memory state of SFB-induced IL-17A and/or IL-22 producing CD4+ T cells based on the expression of specific markers including CCR6, CD44, and CD62L in the presence or absence of *Salmonella* infection (**Figure 4A**). We observed that regardless of the infection state in SFB colonized eSPF mice, within small intestinal LPLs nearly 100% of IL-17A+IL-22- cells expressed CD44+ and lacked CD62L expression with around 70% of them expressing CCR6, which is in contrast to IL-17A-IL-22- cells (**Figure 4A**). Strikingly, after *S. tm.* infection, in cecal LPLs of SFB colonized eSPF mice IL-17A+IL-22+, IL-17A+IL-22-, and IL-17A-IL-22+ cells demonstrated a similar phenotype as small intestinal IL-17A+IL-22- cells (**Figure 4A**). Notably, fewer IL-17A-IL-22+ CD4+ T cells expressed CCR6 compared to other cytokine releasing cell subsets in the cecum. These findings were further corroborated by isolation and characterization of the cell subsets from SFB-monocolonized GF mice (**Supplementary Figure S4A**) suggesting a memory or tissue-resident CD4+ T cell phenotype of these cells.

Next, we wanted to characterize the gene expression profile of distinct *in vivo* cytokine producing cells comparing antigen-specific and bystander activation by sorting these cells based on their cytokine expression directly *ex vivo*. From SFB colonized eSPF mice, we FACS sorted three distinct populations of CD3+CD4+ T cells: IL-17A+IL-22- cells from small intestinal LPLs of uninfected mice and IL-17A-IL-22+ and IL-17A+IL-22+ cells from cecal LPLs of *S. tm.* infected mice (**Supplementary Figure S4B**). The purity of the different subsets of FACS sorted cells was confirmed using specific quantitative PCR for *Il17a* and *Il22* gene expression (**Supplementary Figure S4C**). The comparison of gene expression revealed a significant difference between conventional Th17 cells (IL-17A+IL-22-) from the small intestine and bystander activated Th17/22 cells (IL-17A+IL-22+) from the cecum (**Figure 4B**). Specifically, we observed more than 2,000 genes were differentially up- or downregulated (FC > 2, p < 0.05) (**Figure 4C**). Numerous cellular process and pathways likely reflecting the different activation status were affected.

Next, we compared the gene expression of bystander activated IL-17+IL-22+ and IL-17-IL-22+ CD4+ T cells. Although PCoA analysis revealed a distinct clustering (**Figure 4D**), the number of differentially expressed genes (FC > 2, p < 0.05) between these two groups was considerably lower than between Th17 and Th17/22 (99 vs 2,087 genes respectively) (**Figure 4E**). Besides the expected differential expression of *Il17a* and *Il17f* in Th17 (22) cells, we observed a higher expression of *Cxcl3*, a chemokine involved in immune cells migration and adhesion. In contrast, Th22 cells demonstrated, for instance, an increased expression of *Tcf7*, a transcription factor associated with negative regulation of IL-17 expression (37). Moreover, bystander activated IL-17-IL-22+ CD4+ T cells highly expressed Th1 associated genes like *Il12rb2* and *Ifnγ*. Increased expression of *Il17f* by Th17 (22) cells and *Ifnγ* by Th22 cells were confirmed by quantitative PCR (**Supplementary Figure S4D**). Together, these data suggest that IL-17+IL-22+ and IL17-IL-22+ CD4+ T cells differ in the expression of additional cytokines and receptors that may contribute to unique functions of the subpopulations during enteric infections.

### Segmented Filamentous Bacteria-Induced IL-17A^-^IL-22^+^ CD4 T Cells Do Not Share a Cytokine Expression Profile With Other CD4+ T Cells

To effectively exert their effector function, memory CD4+ T cells subsets express distinct sets of cytokines after antigen encounter. Strikingly, diverse Th cell subsets with various combinations of cytokine expression after *ex vivo* restimulation have been described in the human intestine that do not fit into the classical Th cell subsets (21). Whether the microbiota or other antigens prime these subsets is unknown. While some studies have reported an increase of IFN-γ and IL-10 production from CD4+ T cells after SFB colonization as well the fundamental participation on the induction of systemic T follicular helper (Tfh) cells (38, 39), Littman and colleagues have reported that SFB induces exclusively Th17 cells that maintain their cytokine expression profiles even in conditions of Th1 polarization (24).

**Figure 5:**
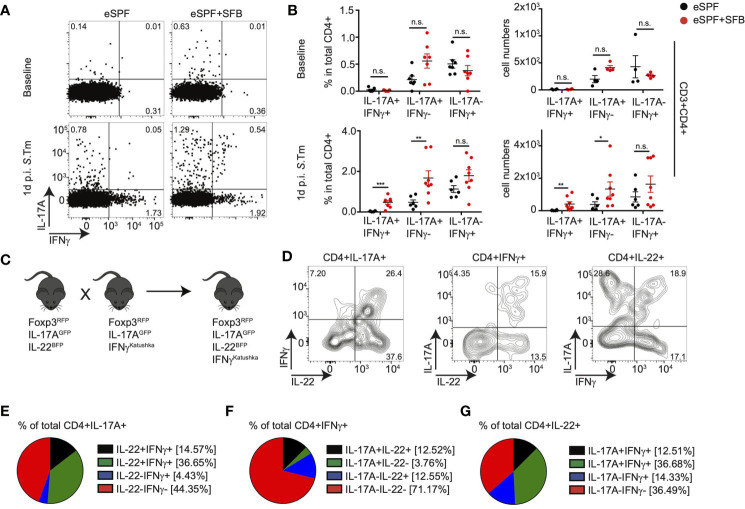
IL-22 producing CD4 T cells are induced by SFB independent of IFNγ+ CD4 T cells. **(A, B)** LPL were isolated from baseline and 1d post *S. tm*. infection from the cecum of IL-17A^GFP^ IFN-γ^Katushka^ FoxP3^RFP^ mice harboring eSPF or eSPF+SFB. Representative FACS plots **(A)** and percentages and absolute numbers **(B)** of CD3+CD4+ cells expressing IL-17A and IFN-γ in the absence of *ex vivo* restimulation. **(C)** A quadruple knock-in reporter mice were derived by crossing IL-17A^GFP^ IL-22^BFP^ FoxP3^RFP^ and IL-17A^GFP^ IFN-γ^Katushka^ FoxP3^RFP^ mouse lines. **(D–G)** LPL were isolated from 1d post *S*. *tm.* infection from the cecum of quadruple reporter mice and analyzed by FACS. Representative FACS plots of CD3+CD4+ cells expressing IL-17A, IL-22, and/or IFN-γ in the absence of *ex vivo* restimulation **(D)**. Frequencies of cytokine secretion by CD4 T cells expressing IL-17A **(E)**, IFN-γ **(F)**, or IL-22 **(G)**. Data represent n = 6–9 mice/group as mean ± SEM from at least two independent experiments. P-values indicated represent an unpaired Student’s t test *p < 0,05; **p < 0,01; ***p < 0,001; n.s., not significant. See also **Supplementary Figure S5**.

Since we identified IFN-γ expression from IL-17A+IL-22+ and IL-17A+IL-22+ CD4+ T cells using gene expression analysis, we aimed to analyze whether all SFB-induced Th17 cells co-produce IFN-γ or whether this is potentially specific for certain subpopulations during bystander activation after *Salmonella* infection. Therefore, we utilized IL-17A^GFP^ IFN-γ^Katushka^ FoxP3^RFP^ mice rederived into the eSPF environment (40). In line with our previous observations, we observed IL-17A+ T cells in steady state conditions predominantly in the SI of eSPF+SFB mice, where a subset of them produced also IFN-γ *in situ* (**Supplementary Figures S5A, B**). Early after *Salmonella* infection, IL-17A+IFN-γ- and IL-17A+IFN-γ+ but not IL-17A-IFN-γ+ CD4+ T cells were significantly increased in the cecum of eSPF+SFB compared to eSPF mice (**Figures 5A, B**) suggesting that colonization with SFB results in the expansion of Th17-related subpopulations of CD4+ T cells but not classical Th1 cells. Next, to further extend our analysis to IL-22, we intercrossed the two reporter mouse lines to create a quadruple IL-17A^GFP^ IL-22^BFP^ IFNγ^Katushka^ Foxp3^RFP^ reporter mouse line (**Figure 5C**). FACS analysis of cecal CD4+ T cells after *Salmonella* infection of eSPF+SFB mice revealed that ~70% of IFN-γ producing CD4 T cells did not secrete IL-17A or IL-22 indicating a classical Th1 phenotype (**Figures 5D, F**). The majority of IL-17A was derived from cells producing only IL-17A or IL-17A and IL-22 together, which was similar for IL-22, which was derived largely either from cells expressing only IL-22 or IL-17A and IL-22 (**Figures 5D, E, G**). Notably, almost all possible combinations were routinely detected, except cells that were IL17A+IL-22-IFN-γ+ (**Figures 5D–G**). Together, these data demonstrate that SFB does not affect intestinal Th1 cell abundances but that SFB rather induces CD4+ T cells with diverse cytokine secretion profiles including a subset of cells producing only IL-22 upon enteric infection.

### Segmented Filamentous Bacteria-Induced Th22 Cells Require Distinct Cytokine Stimulation for Its Effector Function

**Figure 6:**
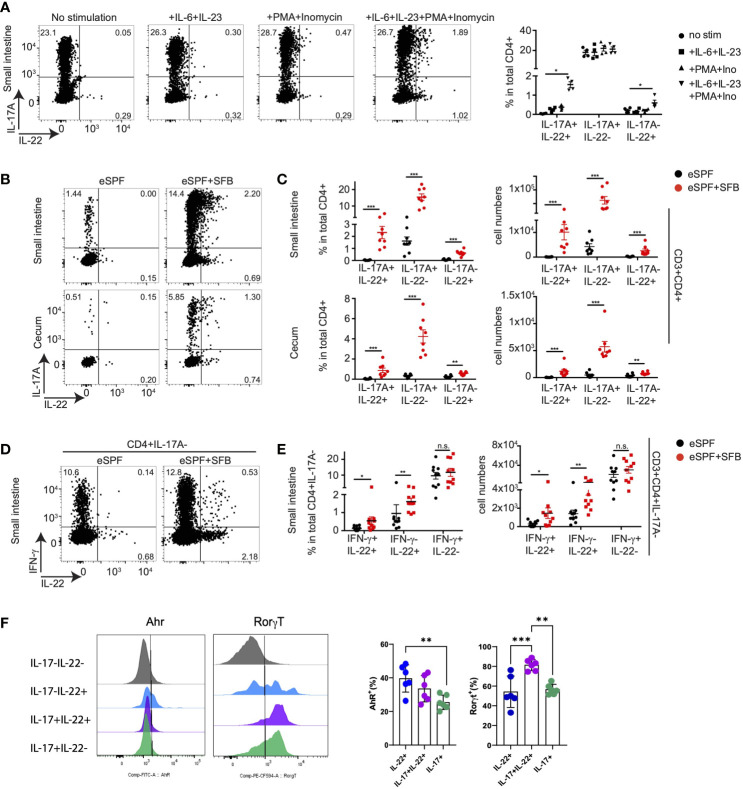
SFB induced CD4 T cells during steady state require cytokine stimulation to secret IL-22. **(A)** Small intestinal LPL were isolated from baseline of IL-17A^GFP^ IL-22^BFP^ FoxP3^RFP^ mice harboring eSPF+SFB. Isolated LPL were stimulated for 5hrs upon indicated conditions and analyzed by FACS. Left, representative FACS plots and right, frequencies of CD3+CD4+ cells expressing IL-17A and IL-22. **(B, C)** LPL were isolated from baseline of IL-17A^GFP^ IL-22^BFP^ FoxP3^RFP^ mice harboring eSPF or eSPF+SFB. Isolated LPL were stimulated for 5hrs in presence of IL-6, IL-23, PMA and ionomycin and analyzed by FACS. Left, representative FACS plots **(B)** and right, frequencies **(C)** of CD3+CD4+ cells expressing IL-17A and IL-22. **(D, E)** LPL were isolated from the baseline of non-reporter (WT) mice harboring eSPF or eSPF+SFB. Isolated LPL were stimulated for 5 h in presence of IL-6, IL-23, PMA, and ionomycin and analyzed by FACS for intracellular cytokine production. Left, representative FACS plots **(D)** and right, frequencies **(E)** of CD3+CD4+IL-17A- cells expressing IFN-γ and IL-22. **(F)** LPL were isolated from the baseline of non-reporter (WT) mice harboring eSPF+SFB. Isolated LPL were stimulated for 5 h in the presence of IL-6, IL-23, PMA, and ionomycin and analyzed by FACS for transcription factor and cytokine production. Left, representative FACS plots gated on the indicated subsets of CD4+ T cells and right, frequencies of transcription factor positive cells within the indicated subsets. Data represent n = 4–8 mice/group as mean ± SEM from at least two independent experiments. P-values indicated represent an unpaired Student’s t test *p < 0,05; **p < 0,01; ***p < 0,001; n.s., not significant. See also **Supplementary Figure S6**.

IL-22 producing CD4+ T cells have been identified in diverse tissues including the intestine (15, 41, 42). The increased abundance of IL-17A-IL-22+ cells CD4+ T cells after *Salmonella* infection of eSPF+SFB compared to eSPF mice triggered the question of whether these SFB-induced cells are also detectable in steady state conditions. Yet, *in vitro* restimulation of CD4+ T cells from eSPF and eSPF+SFB mice with PMA and ionomycin resulted in the production of IL-22 from only a small fraction of CD4+ T cells (0.22% +/- 0.09) (**Figure 6A**). Since *Salmonella* infection results in a massive cytokine storm in the intestinal mucosa, we hypothesized that infection-induced cytokines would potentially be required to efficiently stimulate IL-22 production from Th22 or related CD4+ cell subpopulations. Notably, both IL-6 and IL-23 have already been reported to induce IL-17 and IL-22 secretion *in vitro* from CD4 T cells (43, 44). Hence, isolated LPL from IL-17A^GFP^ IL-22^BFP^ Foxp3^RFP^ reporter mice harboring the eSPF+SFB microbiota were restimulated for 5hrs under different conditions (**Figures 6A** and **Supplementary Figure S6A**). Strikingly, stimulation of cells with IL-6 and IL-23 together with PMA and ionomycin resulted in an increased production of IL-22 in both IL-17+ and IL-17- CD4+ T cells (**Figure 6A**). IL-6 and IL-23 alone were not sufficient to efficiently stimulate IL-22 production. Moreover, IL-17 production alone was not affected under this condition (**Figure 6A**). Next, CD4+ T cells from the SI and cecum from eSPF and eSPF+SFB mice were stimulated in optimized conditions to assess whether SFB induces a subpopulation of cells producing IL-22 but not IL-17A *ex vivo* in the steady state. Strikingly, a significant increase in IL-22 production was observed only from CD4+ T cells in SFB colonized mice (**Figures 6B, C**). Moreover, unlike *Salmonella* infection CD4+ T cells from the SI also released IL-22 upon *ex vivo* restimulation suggesting that these cells also reside in the SI (**Figures 6B, C**). In contrast, CD4+ T cells from the mesenteric lymphnodes from the SFB colonized mice did not demonstrate any significant IL-22 producing ability (**Supplementary Figures S6B, C**). Since our earlier findings indicated the presence of subpopulations of IFN-γ and IL-22 co-producing CD4+ T cells, we tested whether SFB can induce CD4+ T cells secreting only IL-22. To this end, we characterized the expression of IL-17A, IFN-γ and IL-22 from small intestinal LPL isolated from eSPF and eSPF+SFB colonized mice during a steady state and restimulated under optimized conditions. Strikingly, we observed a significant increase in IL-22 single and IFN-γ co-producing CD4+IL-17A- T cells in mice colonized with SFB (**Figures 6D, E**). In line with our previous findings, CD4+ T cells producing only IFN-γ were not affected by the presence or absence of SFB (**Figures 6D, E**).

We next performed analyzed transcription factor expression in IL-22- and IL-17-producing CD4+ T cells. Our initial analysis of LPLs from IL-17A^GFP^ IL-22^BFP^ Foxp3^RFP^ reporter mice failed to detect expression of the cytokines from FoxP3+ cells (data not shown). Next, ileal LPLs from mice harboring the eSPF+SFB microbiota were next restimulated for 5hrs with PMA, ionomycin, IL-6, and IL-23 followed by staining for cytokines (IL-17A and IL-22) and transcription factors (RORγt, Ahr) (**Figure 6F**). The analysis demonstrated that within the IL-22+IL17- CD4+ T cell subset, approx. 40% expressed Ahr and RORgt. From the IL-17+IL-22+ CD4+ T cells, the large majority were RORgt+ and only a minority expressed Ahr, i.e., 81% and 33%, respectively. While these analyses do not clearly identify the exclusive expression patterns between the subpopulations, specific differences could be observed. Together, these data demonstrate that SFB colonization induces a CD4+ T cell population producing IL-22 but not IL-17A or IFN-γ, which is also present under steady state conditions. They may represent a Th22 cell subset that releases IL-22 upon encountering specific cytokine signals in addition to TCR stimulation.

### Segmented Filamentous Bacteria-Induced Th22 Cells Develop Independent of IL-17 Secretion and TGF-β Signaling

**Figure 7:**
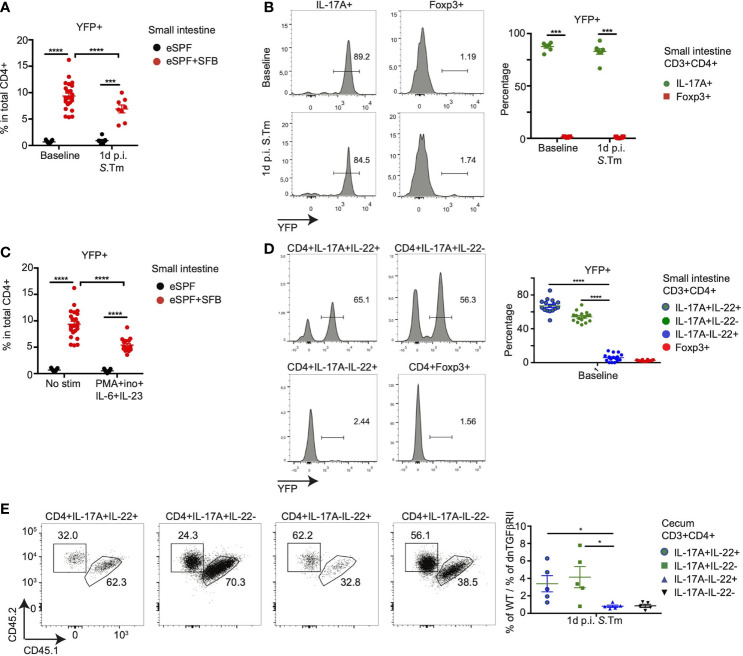
SFB-induced Th-22 cells development is independent of IL-17 secretion. **(A, B)** CD3+CD4+ T cells from small intestinal LPL from eSPF and eSPF+SFB colonized IL-17A^CRE^ R26^YFP^ IL-17A^GFP^ IL-22^BFP^ Foxp3^RFP^ (Fate reporter) mice were analyzed for YFP expression during baseline and 1d post *S*. *tm*. infection without *ex vivo* restimulation. YFP expression in total CD3+CD4+ cells **(A)**. YFP expression in IL-17A+ and Foxp3+ cells from eSPF+SFB colonized mice **(B)** and in left, representative FACS plots and right, frequencies of YFP+ cells in CD4 T cells expressing IL-17A and Foxp3. **(C, D)** CD3+CD4+ T cells from small intestinal LPL from eSPF and eSPF+SFB colonized Fate reporter mice were stimulated *ex vivo* under stated condition and analyzed for YFP expression during baseline. YFP expression in total CD3+CD4+ cells **(C)**. YFP expression in IL-17A+ and/or IL-22+ cells and Foxp3+ cells from eSPF+SFB colonized mice **(D)** and in left, representative FACS plots and right, frequencies of YFP+ cells in CD4 T cells expressing IL-17A and/or IL-22 and Foxp3 without any *ex vivo* restimulation. **(E)** CD3+CD4+ T cells from cecal LPL after 1d *S*. *tm.* infection from eSPF+SFB colonized *Rag2^-/-^* mice, that received CD4+ T cells from congenically labeled WT and CD4dnTGFβRII mice (1:1), were analyzed for expression of congenic markers in IL-17A+ and/or IL-22+ and Foxp3+ cells after restimulation. Left, representative FACS plots and right, frequencies of YFP+ cells in CD4 T cells expressing IL-17A and/or IL-22 and Foxp3. Data represent n = 5–17 mice/group as mean ± SEM from at least two independent experiments. P-values indicated represent an unpaired Student’s t test *p < 0,05; ***p < 0,001; ****p < 0,0001. See also **Supplementary Figure S7**.

The concept of plasticity of the Th cell subsets has emerged to describe the conversion of one of the classical subsets of Th cells into another, e.g., the transdifferentiation of Th17 into T regulatory type 1 cells has been recently identified (40, 45). In order to address whether the subpopulation of IL-17-IL22+ CD4+ T cells derive from IL-17A producing cells in the SI, we utilized an IL-17 fate reporter system in which enhanced yellow fluorescent protein (YFP) permanently labels all cells that have temporarily expressed IL-17A (IL-17A^CRE^ R26^YFP^) (40, 46). We crossed these mice with IL-17A^GFP^ IL-22^BFP^ Foxp3^RFP^ reporter mice to obtain IL-17A^CRE^ R26^YFP^ IL-17A^GFP^ IL-22^BFP^ Foxp3^RFP^ mice, which allow to identify whether IL-22 producing CD4+ T cells in the SI and cecum derive from cells that have formerly expressed IL-17A. Colonization of these mice with SFB induced the expression of YFP in 9% of CD4+ T cells, while in eSPF mice, only 0.6% were positive demonstrating that YFP+ cell are almost exclusively induced by SFB (**Figure 7A**). Nearly all CD4+ T cells producing IL-17A *in situ* in the SI of mice colonized with SFB expressed YFP (85%), which was absent from FoxP3+ T cells (**Figure 7B**). In line with our previous observations, no CD4+ T cells producing IL-22 *in situ* were observed, hence, LPL were restimulated under optimized conditions, which did not result in an increased YFP expression in CD4+ T cells itself (**Figure 7C**). While YFP expression was observed in a large fraction of IL-17A+ (54%) and IL-17+IL-22+ (67%) CD4+ T cells, IL-22+IL17A- CD4+ T cells expressed almost no YFP (**Figure 7D**). This indicates that SFB-induced IL-22+IL-17A- CD4+ T cells are likely not derived from an exTh17 population in the SI. Although YFP+ CD4+ T cells were substantially low in cecal LPL (**Supplementary Figure S7A**), similar to the restimulation of cells from the SI, IL-17-IL-22+ CD4+ T cells from the cecum after *Salmonella* did express low levels of YFP comparable to Foxp3+ CD4 T cells, while half of the IL-17+ cells expressed YFP (**Supplementary Figure S7B**). This strongly suggests that IL-22+IL-17A- CD4+ T cells do not derive from IL-17A+ population.

We next questioned whether IL-22-producing cells develop independently from signals important for Th17 cell development. Hence, we addressed the role of transforming growth factor (TGF)-β, which plays an essential role for Th17 differentiation in both mouse models and human (31, 47, 48). To investigate whether SFB induced Th17 (22) and Th22 cells also require TGF-β signaling for their differentiation, we used transgenic mice expressing a dominant negative form of TGF-β receptor II under control of the *Cd4* promoter (CD4dnTGFβRII mice) (49, 50). We transferred CD4+ T cells from both wild type (CD45.1, CD45.2) and CD4dnTGFβRII (CD45.2) mice in a 1:1 ratio into *Rag2^-/-^* mice harboring the eSPF+SFB microbiota. The recipient mice were infected with *S. tm.* after 4 weeks. Conventional cells from the small intestinal LPL showed an enrichment of Th17 cells from the wild type donor confirming the importance of TGF-β signaling for Th17 differentiation (**Supplementary Figure S7C**). Similar to our previous results, LPL isolated from the cecum of the *Rag2^-/-^* recipient mice contained IL-17A and IL-22 producing CD4+ T cells (data not shown). Similar to small intestinal Th17 cells, cecal Th17 and Th17 (22) cells were significantly enriched with cells from the WT donor (**Figure 7E**). In contrast, IL-17A-IL22+ CD4 T cells had developed even in the absence of TGF-β signaling (**Figure 7E**).

Together, these data demonstrate that SFB induces a subpopulation of CD4+ T cells with the ability to release IL-22 but not IL-17, which are not derived from IL-17-producing Th17 cells in the SI and unlike Th17 cells develop independent of TGF-β signaling suggesting that they are potentially bona fide Th22 cells.

## Discussion

In the last decade, researchers have started to recognize that specific members of the intestinal microbiota have a disproportional influence on the development and function of immune cell subsets in the mucosa and beyond. Hence, understanding the contribution of specific members of the microbiota on immune homeostasis and inflammation has emerged as a central question in many physiological and inflammatory processes, including mucosal infections. Here, we demonstrate that colonization with SFB, a well-recognized *de novo* inducer of Th17 cells, results in an enhanced numbers of T cells with the potential to produce IL-22 but not IL-17. Moreover, we showed that these IL-22+IL-17- T cells, presumably Th22, develop independent of the signals required for classical Th17 differentiation and release these cytokines in the mucosa early during enteric *Salmonella* infection.

IL-22 plays an important role in the cross-talk between immune cells and non-immune cells in several tissues regulating tissue regeneration and anti-microbial immunity. Multiple cellular sources of IL-22 have been identified including ILC3s, γδ T cells and CD4+ T cells. Within the CD4+ T cells, multiple subsets including Th17 and Th1 cells have been identified as sources of IL-22 production. Additionally, the existence of a distinct subset of Th22 has been proposed, yet, its status is still being debated largely due to an overlap between IL-22 production with lineage signature cytokines such IL-17A and IFNγ. The hypothesis that potentially multiple subsets of CD4+ T cell including Th1, Th17, and Th22 cells are a source of IL-22 *in vivo* has been supported by the fate-mapping of IL-22 producing cells during inflammation models (51). These experiments revealed that many cells that produced IL-22 during their lifetime lost the ability to produce IL-22 or also acquired production of cytokines such as IL-17 or IFN-γ (51). Moreover, evidence of plasticity for these cell lineages both *in vivo* and *in vitro* depending on types of inflammation and culture conditions have been reported, respectively (44, 46, 52). Yet, many of these experiments required the restimulation of immune cells *ex vivo*, which has already been demonstrated in other experimental settings not to reflect *in vivo* cytokine production capabilities (53). Hence, to investigate the relation between IL-17A and IL-22 production in CD4+ T cells *in vivo*, we decided to utilize a novel IL-22^BFP^ reporter allele (31) crossed with a previously developed IL-17AGFP reporter allele. We assessed cytokine production in the steady state and during early phases of enteric inflammation at which time CD4+ T cells have been demonstrated to produce IL-17 and IL-22 after bystander activation (26). In line with these previous reports that utilized the restimulation of T cells to measure cytokine production, CD4+ T cells produced little IL-22 in the steady state, but already at early time points after infection they are a significant source of IL-22, even in the absence of *ex vivo* restimulation. Memory or tissue-resident CD4+ T cells, identified by the expression of CD62L and CD44, are the major source of IL-17A and IL-22 cytokine production at this time point. In line with previous work, we could observe an increase in the number of these innate-like types of IL-17A+IL-22- and IL-17A+IL-22+ CD4+ T cells in cecal LPL from infected mice (26). Moreover, we also observed a significant increase in the numbers and frequencies in IL-17A-IL-22+ CD4+ T cells. However, in a steady state condition, our finding contrasted previous report of IL-22 reporter mice, since we did not detect any IL-22 production from intestinal LPLs from non-infected mice (54). This may reflect differences in microbiota and diet composition in animal colonies, since these factors have been demonstrated to modulate IL-22 production. Notably, IL-22 is not detectable in the colonic mucosa of healthy human subjects; however, IL-22 expression is readily detectable from CD4+ T cells in the colonic mucosa of IBD patients (55). Studies have demonstrated that the frequency of IL-22-producing cells is increased in UC patients as well as CD patients, indicating a possible pro-inflammatory role in the etiology of IBD or an attempt of the cells to contribute to tissue repair (55, 56). To investigate the involvement of specific microbiota modulating such innate-like CD4+ T cells, we rederived our reporter mice into an eSPF barrier lacking many known potential pathobionts i.e., SFB, *Helicobacter* spp., Prevotellaceae, murine Norovirus, and protozoa that have previously been reported to modulate host adaptive immunity (28). Strikingly, mice from this eSPF barrier almost completely lost their ability to induce innate IL-17A and IL-22 producing CD4+ T cells upon *Salmonella* infection and cytokine production ability was successfully restored upon colonization with SFB. Notably, at early stages of *Salmonella* infection enhanced numbers of CD4+ T cells producing only IL-22 are observed in SFB colonized mice, which to our knowledge is the first report to observe microbiota modulation of Th22 cells in mice. Under steady state conditions only classical IL-17A producing Th17 were observed in small intestinal LPLs suggesting that IL-22 production is not directly linked to IL-17 production, either stemming from a different subset of cell or requiring different induction signals.

In order to characterize the interrelation between IL-17A-IL-22+ and IL-17+ CD4+ T cells, we performed several additional analyses. First, analysis of Vβ14 TCR enrichment, a TCR chain enriched in CD4 T cells specific for SFB (24), showed a comparably increased ratios in IL-17A-IL-22+ and IL-17+IL-22+ CD4+ T cells compared to other subsets suggesting that they may recognize similar SFB-derived antigens. However, these experiments fall short of directly demonstrating antigen specificity and require further experimentation using SFB-derived antigens and full TCR analysis on the level of single cells rather than only analyzing specific TCR chains in bulk populations. Second, cytokine fate mapping is a sophisticated method extensively used in recent years to described T cell plasticity in different studies (40, 45). Using IL-17 fate mapping mice, we demonstrated that SFB induced Th22 cells have not secreted IL-17A during their development indicating a distinct development of these cell subsets compared to classical SFB-induced Th17. This hypothesis is further supported by a third analysis, i.e., of mice with altered TGF-β signaling, demonstrating that SFB induced Th22 develop independently of TGF-β signaling which is, in contrast, required for Th17 cells. Of note, similar observations have been made for IL-22-producing CD4+ T cells in colitis-associated colon cancer (31). Together, these findings suggest that *in vivo* SFB is not only essential to induce steady-state Th17 cells in the small intestine, but also provide signals resulting in the development of CD4+ T cell subsets characterized by the distinct combinations of IL-22 and IL-17A production rapidly after infection in the intestine.

To complement the characterization of SFB-induced rapid or “innate-like” CD4+ T cells based on their cytokine production, we employed a transcriptional profiling of these distinct cell subsets directly isolated from intestinal LPLs without any restimulation. Strikingly, IL-17A+IL-22+ CD4+ T cells isolated from *S*. *tm.* infected mice demonstrated distinct gene expression profiles compared to ileal Th17 cells isolated from the SI of SFB colonized mice at baseline and clustered more closely to IL-22 only producing CD4+ T cells. Strikingly, these differences were mainly due to the significantly increased expression of *Ifnγ* and *Il17f* by IL-17A-IL-22+ and IL-17A+IL-22+ CD4+ T cells compared to ileal Th17 cells. Increases in *Ifnγ* expression might benefit the host *via* diverse mechanisms such as controlling pathogen loads in the intestinal tissue and regulating mucin release by goblet cells or other so far unknown effects (57–59). *Ifnγ* expression by Th22 subsets questioned whether SFB induces Th1 cells that are able to secret IL-22 upon infection. Previous reports regarding the role of SFB to induce IFN-γ producing CD4+ T (Th1) cells were contradictory demonstrating no Th1 induction to significant Th1 induction in presence of SFB (22, 38). Using a combination of cytokine reporter mice, we demonstrated a direct influence of SFB on IL-22 producing cell subset but not on only IFN-γ−producing cells. Cytokine expression analysis were complemented with an analysis of transcription factors using RNAseq and flow cytometry. For instance, RNAseq analysis revealed only a minor increase in *Tbx21* expression in these innate like CD4+ cells compared to classical Th17 cells. Flow cytometry-based analysis of transcription factors of IL-17A-IL-22+ and IL-17A+IL-22+ CD4+ T cells further identified differences in Ahr and RORγt expression between the two subsets, but without a clearly distinct pattern, i.e., a significant fraction of cells within the subsets share Ahr and RORγt expression. Hence, further detailed studies regarding the transcriptional regulation of these specific Th cell subsets are still necessary, ideally on the level of single cells in combination with TCR analysis to analyze interrelations between the subsets. An additional question that stills remain to be clarified is the role of cytokines in the induction of IL-22 expression in comparison to IL-17. Our experiments have identified that IL-6 and IL-23 assist in IL-22 induction from CD4+ T cells, yet, the contribution of additional cytokine such as IL-1b, which enhances IL-22 production in ILC3s (60), has not been tested.

In conclusion, our study allowed us to to identify a population of CD4+ T cells producing IL-22 and quantitatively assess the effect of *de novo* colonization of SFB on the differentiation of this subset using using gnotobiotic mice. The exact role of adaptive immune cell derived IL-22 during antimicrobial immunity remains to be investigated, including potential reduncies to ILC-derived IL-22 in the induction of antimicrobial peptides and tissue repair. While this and several observations will require additional experiments as discussed above, we believe that this knowledge will eventually allow identifying the sources of pathogenic memory cells that alter disease susceptibility upon bystander activation during inflammatory responses.

## Data Availability Statement

The datasets presented in this study can be found in online repositories. The names of the repository/repositories and accession number(s) can be found in the article/**Supplementary Material**.

## Ethics Statement

All animal experiments were performed in agreement with the local government of Lower Saxony, Germany.

## Author Contributions

UR and TS designed the experiments and wrote the manuscript with input from co-authors. UR, RO, and AG performed and analyzed the experiments. EG supported the analysis of low-input RNA sequencing data. RO, MB, AB, SH, and NG contributed the essential reagents and/or analysis. TS supervised the study. All authors contributed to the article and approved the submitted version.

## Funding

The project was supported by the Helmholtz Association (VH-NG-933 to TS), by the DFG (STR-1343/1 to TS), the EU (StG337251 to SH) and the ERC (CoG 865466 to SH).

## Conflict of Interest

The authors declare that the research was conducted in the absence of any commercial or financial relationships that could be construed as a potential conflict of interest.

## Publisher’s Note

All claims expressed in this article are solely those of the authors and do not necessarily represent those of their affiliated organizations, or those of the publisher, the editors and the reviewers. Any product that may be evaluated in this article, or claim that may be made by its manufacturer, is not guaranteed or endorsed by the publisher.

## Supplementary Material

Supplementary Figure S1 CD4+ T cells contribute to mucosal IL-17A and IL-22 secretion upon bacterial infection **(A)** Experimental procedure for infection with Salmonella enterica serovar Typhimurium (S. Tm.), briefly Streptomycin-pretreated mice were orally infected with 105 of S. Tm. Lamina propria leukocytes (LPL) were isolated from small intestine of non-infected (baseline) and 1 day after S. Tm. (S. Tm. 1d p. i.) infected IL-17A^GFP^ IL-22^BFP^ Foxp3^RFP^ (conventionally raised) mice and analyzed by FACS without any *ex vivo* restimulation. **(B)** Gating strategy of FACS data is displayed. **(C–D)** Representative FACS plots showing IL-17A and IL-22 frequencies gated on total CD3+CD4+ cells **(C)** and relative frequencies of indicated cell subsets **(D)**. Data represent n=4-10 mice/group as mean ± SEM from at least two independent experiments. P values indicated represent a unpaired Student’s t test *p < 0,05; **p < 0,01; ***p > 0,001; ****p > 0,0001.

Supplementary Figure S2 Anti-bacterial IL-17A and IL-22 responses by CD4+ T cells are dependent on SFB **(A)** Experimental procedure: eSPF RORγt^GFP^ FoxP3^RFP^ or IL-17A^GFP^ IL-22^BFP^ FoxP3^RFP^ mice were cohoused with SFB monocolonized mice for 3 weeks followed by S. Tm. infection described in **Supplementary Figure S1**. **(B–C)** LPL were isolated during baseline from small intestine and cecum of IL-17A^GFP^ IL-22^BFP^ FoxP3^RFP^ mice harboring eSPF or eSPF+SFB. Representative FACS plots **(B)** and percentages and absolute numbers **(C)** of CD3+CD4+ cells expressing IL-17A and IL-22 without any *ex vivo* restimulation. Data represent n=5-13 mice/group as mean ± SEM from at least two independent experiments. P values indicated represent a unpaired Student’s t test *p < 0,05; **p < 0,01; ***p < 0,001; ****p < 0,0001.

Supplementary Figure S3 Anti-bacterial IL-17A and IL-22 responses by CD4+ T cells are SFB specific **(A–B)** LPL were isolated during baseline from small intestine and cecum of IL-17A^GFP^ IL-22^BFP^ FoxP3^RFP^ mice harboring GF or GF+SFB. Representative FACS plots **(E)** and percentages and absolute numbers **(F)** of CD3+CD4+ cells expressing IL-17A and IL-22 without any *ex vivo* restimulation. **(C)** CD3+CD4+ T cells from small intestinal LPL 1d after S. Tm. infection from GF+SFB were analyzed for Vβ14 expression in IL-17A+ and Foxp3+ cells. Left, representative FACS plots and right, specific enrichment of Vβ14 TCRs in CD4 T cells expressing IL-17A and Foxp3 without any *ex vivo* restimulation. **(D)** CD3+CD4+ T cells from cecal LPL 1d after S. Tm. infection from GF+SFB were analyzed for Vβ14 expression in IL-17A and/or IL-22 and Foxp3+ cells. Left, representative FACS plots and right, specific enrichment of Vβ14 TCRs in CD4 T cells expressing IL-17A and/or IL-22 and Foxp3 without any *ex vivo* restimulation. Data represent n=4-10 mice/group as mean ± SEM from at least two independent experiments. P values indicated represent a unpaired Student’s t test *p < 0,05; **p < 0,01; ***p < 0,001; ****p < 0,0001.

Supplementary Figure S4 Distinct properties of SFB modulated CD4 T cells **(A)** CD3+CD4+ T cells from small intestinal and cecal LPL from GF+SFB mice during baseline and after 1d S. Tm. infection were analyzed for their effector memory (CD44hiCD62Llo) properties and CCR6 expression in IL-17A+ and/or IL-22+ and IL-17A-IL-22- cells in absence of any *ex vivo* restimulation. **(B)** Gating strategy demonstrating sorting of cytokine producing CD3+CD4+ LPL isolated from non-infected and infected eSPF+SFB colonized mice. **(C–D)** Quantitative PCR to detect relative expression of specific gene of interest from cytokine producing CD3+CD4+ cell subsets sorted in S4B. The cells were not restimulated. Data represent n=4-6 mice/group as mean ± SEM from at least two independent experiments. P values indicated represent a unpaired Student’s t test *p < 0,05; **p < 0,01; ***p < 0,001; ****p < 0,0001.

Supplementary Figure S5 IL-22 producing CD4 T cells are induced by SFB independent of IFNg+ CD4 T cells **(A–B)** LPL were isolated from baseline and 1d post S. Tm. infection from small intestine of IL-17A^GFP^ IFN-γ^Katushka^ FoxP3^RFP^ mice harboring eSPF or eSPF+SFB. Representative FACS plots **(A)** and percentages and absolute numbers **(B)** of CD3+CD4+ cells expressing IL-17A and IFN-γ without any *ex vivo* restimulation. Data represent n=6-9 mice/group as mean ± SEM from at least two independent experiments. P values indicated represent a unpaired Student’s t test *p < 0,05; **p < 0,01; ***p < 0,001; ****p< 0,0001.

Supplementary Figure S6 SFB induced CD4 T cells during steady state require cytokine stimulation to secret IL-22 **(A)** Cecal LPL were isolated from baseline of IL-17A^GFP^ IL-22^BFP^ FoxP3^RFP^ mice harboring eSPF+SFB. Isolated LPL were stimulated for 5hrs upon indicated conditions and analyzed by FACS. (B–C) Leukocytes were isolated from mesenteric lymphnode from IL-17A^GFP^ IL-22^BFP^ FoxP3^RFP^ mice harboring eSPF or eSPF+SFB. Isolated LPL were stimulated for 5hrs in presence of IL-6, IL-23, PMA and inomycin and analyzed by FACS. Left, representative FACS plots **(B)** and right, frequencies **(C)** of CD3+CD4+ cells expressing IL-17A and IL-22. Data represent n=4-5 mice/group as mean ± SEM from at least two independent experiments. P values indicated represent a unpaired Student’s t test *p < 0,05; **p < 0,01; ***p< 0,001; ****p < 0,0001.

Supplementary Figure S7 SFB induced Th-22 cells development is independent of IL-17 secretion **(A–B)** CD3+CD4+ T cells from cecal LPL from eSPF and eSPF+SFB colonized Fate reporter mice were isolated 1d post S. Tm infection and analyzed for YFP expression during baseline. YFP expression in total CD3+CD4+ cells **(A)**. YFP expression in IL-17A+ and/or IL-22+ cells and Foxp3+ cells from eSPF+SFB colonized mice **(B)** and in left, representative FACS plots and right, frequencies of YFP+ cells in CD4 T cells expressing IL-17A and/or IL-22 and Foxp3 without any *ex vivo* restimulation. **(C)** CD3+CD4+ T cells from small intestinal LPL after 1d S. Tm. infection from eSPF+SFB colonized Rag2^-/-^ mice, that received CD4+ T cells from congenically labeled WT and CD4dnTGFβRII mice (1 : 1), were analyzed for expression of congenic markers in IL-17A+ and Foxp3+ cells. Left, representative FACS plots and right, frequencies of YFP+ cells in CD4 T cells expressing IL-17A and/or IL-22 and Foxp3 without any *ex vivo* restimulation. Data represent n=5-8 mice/group as mean ± SEM from at least two independent experiments. P values indicated represent an unpaired Student’s t test *p < 0,05; **p < 0,01; ***p< 0,001; ****p< 0,0001.
