# Supplementary figures and images for: Induction of IL-22-Producing CD4+ T Cells by Segmented Filamentous Bacteria Independent of Classical Th17 Cells
Source: Front Immunol. 2021 Sep 8;12:671331. doi: 10.3389/fimmu.2021.671331 (PMC8456099; doi:10.3389/fimmu.2021.671331)

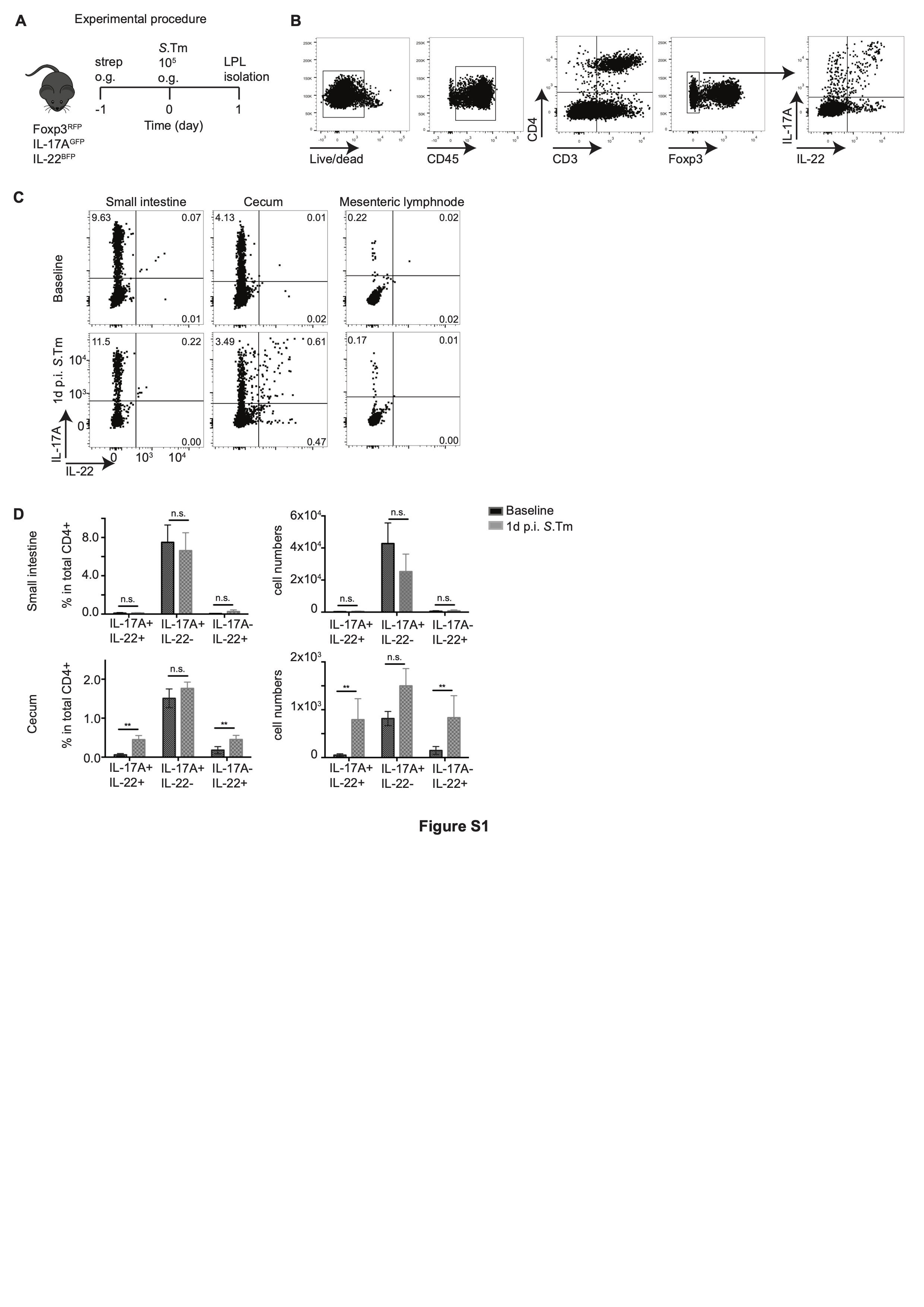

Supplement: Supplementary Figure S1 — CD4+ T cells contribute to mucosal IL-17A and IL-22 secretion upon bacterial infection (A) Experimental procedure for infection with Salmonella enterica serovar Typhimurium (S. Tm.), briefly Streptomycin-pretreated mice were orally infected with 105 of S. Tm. Lamina propria leukocytes (LPL) were isolated from small intestine of non-infected (baseline) and 1 day after S. Tm. (S. Tm. 1d p. i.) infected IL-17AGFP IL-22BFP Foxp3RFP (conventionally raised) mice and analyzed by FACS without any ex vivo restimulation. (B) Gating strategy of FACS data is displayed. (C–D) Representative FACS plots showing IL-17A and IL-22 frequencies gated on total CD3+CD4+ cells (C) and relative frequencies of indicated cell subsets (D). Data represent n=4-10 mice/group as mean ± SEM from at least two independent experiments. P values indicated represent a unpaired Student’s t test *p < 0,05; **p < 0,01; ***p > 0,001; ****p > 0,0001. [file Image_1.jpeg]

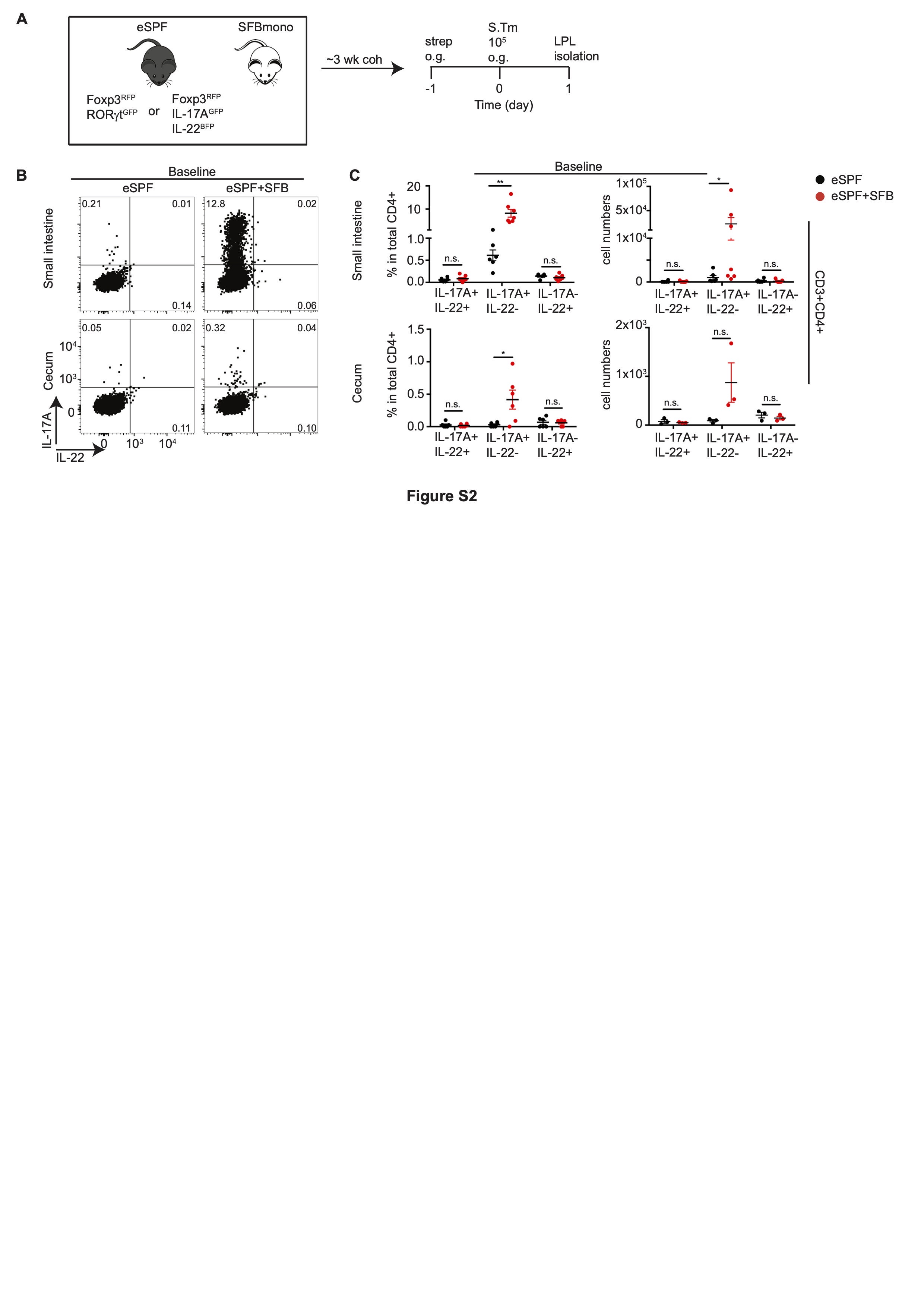

Supplement: Supplementary Figure S2 — Anti-bacterial IL-17A and IL-22 responses by CD4+ T cells are dependent on SFB (A) Experimental procedure: eSPF RORγtGFP FoxP3RFP or IL-17AGFP IL-22BFP FoxP3RFP mice were cohoused with SFB monocolonized mice for 3 weeks followed by S. Tm. infection described in Supplementary Figure S1. (B–C) LPL were isolated during baseline from small intestine and cecum of IL-17AGFP IL-22BFP FoxP3RFP mice harboring eSPF or eSPF+SFB. Representative FACS plots (B) and percentages and absolute numbers (C) of CD3+CD4+ cells expressing IL-17A and IL-22 without any ex vivo restimulation. Data represent n=5-13 mice/group as mean ± SEM from at least two independent experiments. P values indicated represent a unpaired Student’s t test *p < 0,05; **p < 0,01; ***p < 0,001; ****p < 0,0001. [file Image_2.jpeg]

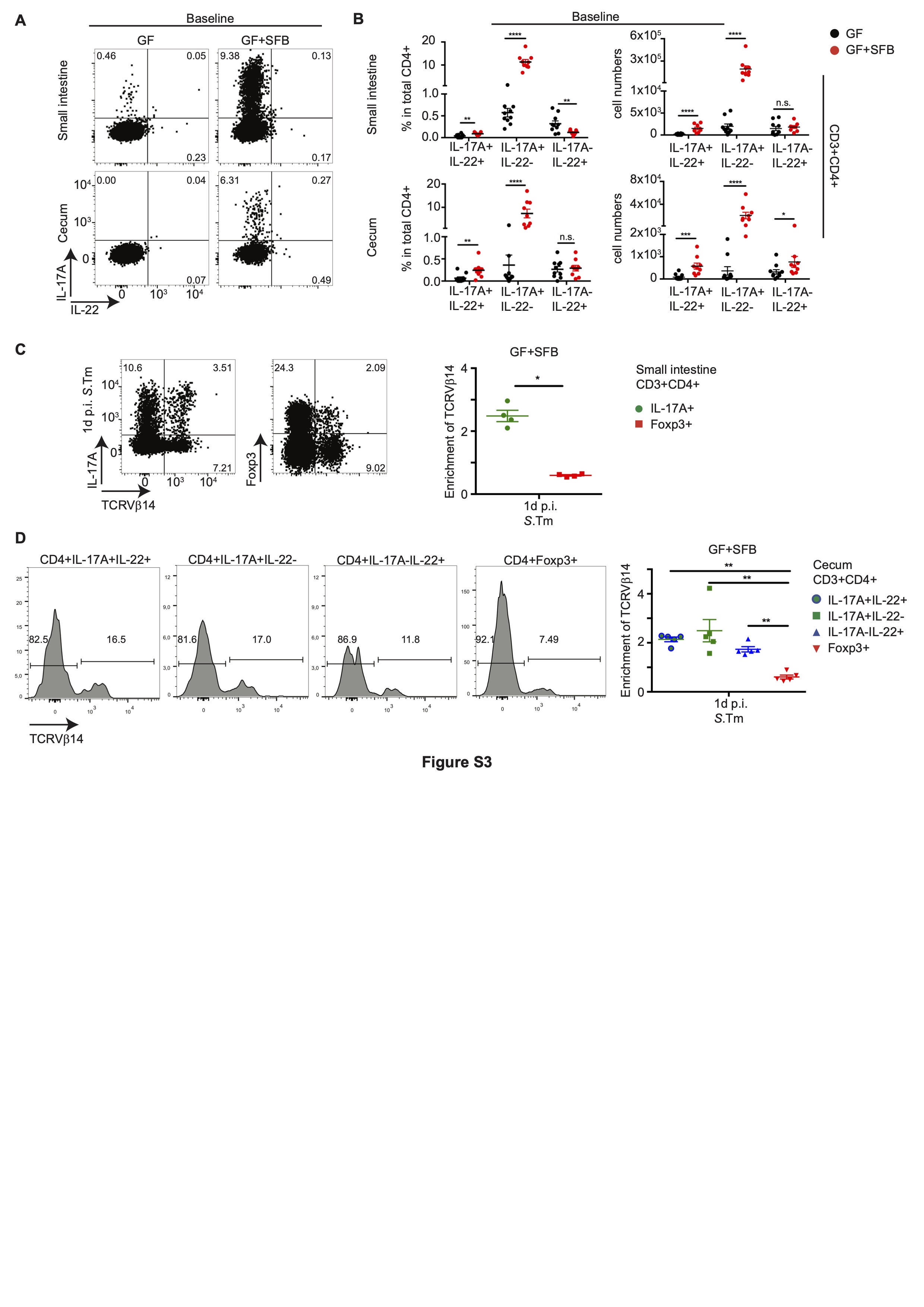

Supplement: Supplementary Figure S3 — Anti-bacterial IL-17A and IL-22 responses by CD4+ T cells are SFB specific (A–B) LPL were isolated during baseline from small intestine and cecum of IL-17AGFP IL-22BFP FoxP3RFP mice harboring GF or GF+SFB. Representative FACS plots (E) and percentages and absolute numbers (F) of CD3+CD4+ cells expressing IL-17A and IL-22 without any ex vivo restimulation. (C) CD3+CD4+ T cells from small intestinal LPL 1d after S. Tm. infection from GF+SFB were analyzed for Vβ14 expression in IL-17A+ and Foxp3+ cells. Left, representative FACS plots and right, specific enrichment of Vβ14 TCRs in CD4 T cells expressing IL-17A and Foxp3 without any ex vivo restimulation. (D) CD3+CD4+ T cells from cecal LPL 1d after S. Tm. infection from GF+SFB were analyzed for Vβ14 expression in IL-17A and/or IL-22 and Foxp3+ cells. Left, representative FACS plots and right, specific enrichment of Vβ14 TCRs in CD4 T cells expressing IL-17A and/or IL-22 and Foxp3 without any ex vivo restimulation. Data represent n=4-10 mice/group as mean ± SEM from at least two independent experiments. P values indicated represent a unpaired Student’s t test *p < 0,05; **p < 0,01; ***p < 0,001; ****p < 0,0001. [file Image_3.jpeg]

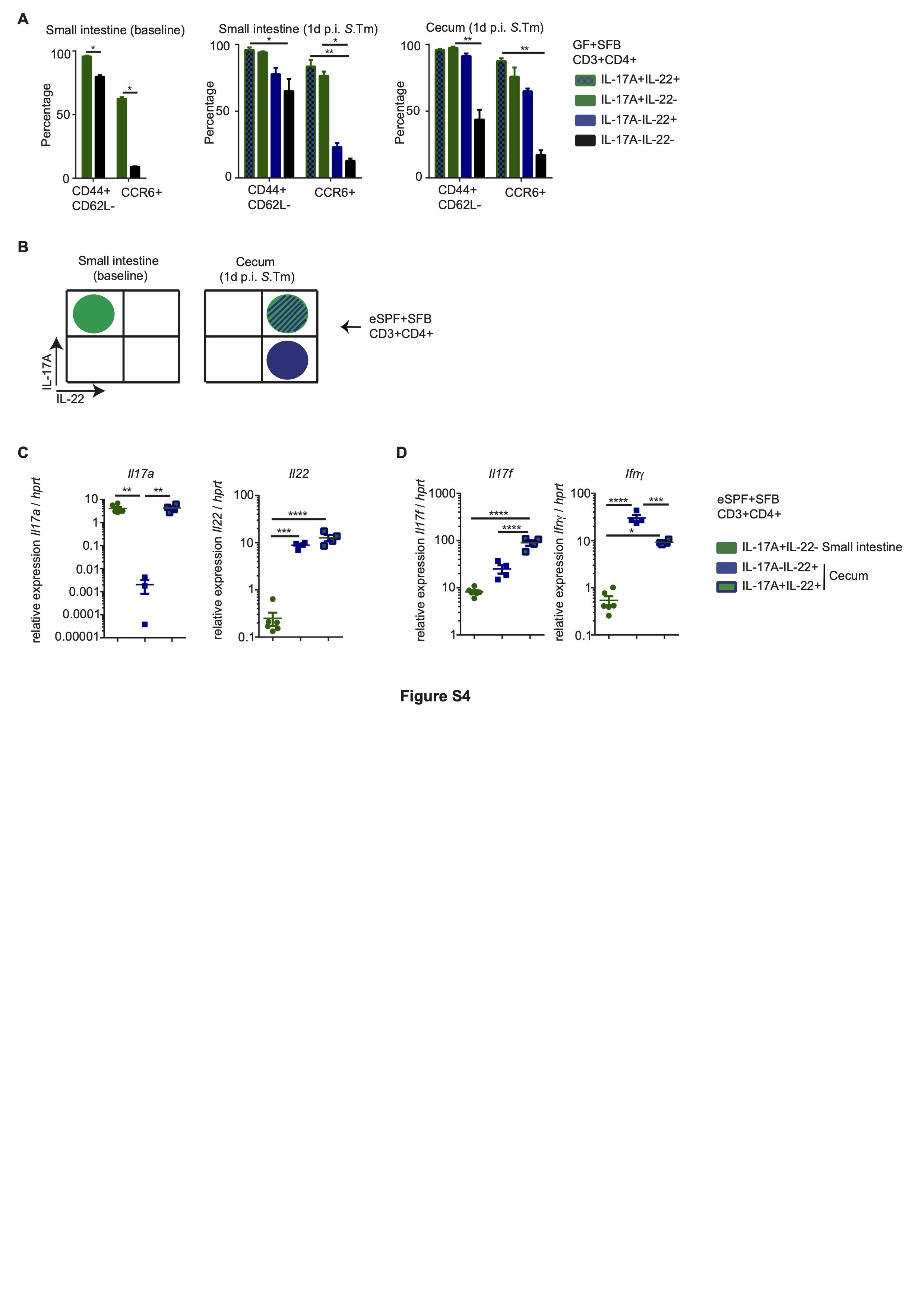

Supplement: Supplementary Figure S4 — Distinct properties of SFB modulated CD4 T cells (A) CD3+CD4+ T cells from small intestinal and cecal LPL from GF+SFB mice during baseline and after 1d S. Tm. infection were analyzed for their effector memory (CD44hiCD62Llo) properties and CCR6 expression in IL-17A+ and/or IL-22+ and IL-17A-IL-22- cells in absence of any ex vivo restimulation. (B) Gating strategy demonstrating sorting of cytokine producing CD3+CD4+ LPL isolated from non-infected and infected eSPF+SFB colonized mice. (C–D) Quantitative PCR to detect relative expression of specific gene of interest from cytokine producing CD3+CD4+ cell subsets sorted in S4B. The cells were not restimulated. Data represent n=4-6 mice/group as mean ± SEM from at least two independent experiments. P values indicated represent a unpaired Student’s t test *p < 0,05; **p < 0,01; ***p < 0,001; ****p < 0,0001. [file Image_4.jpeg]

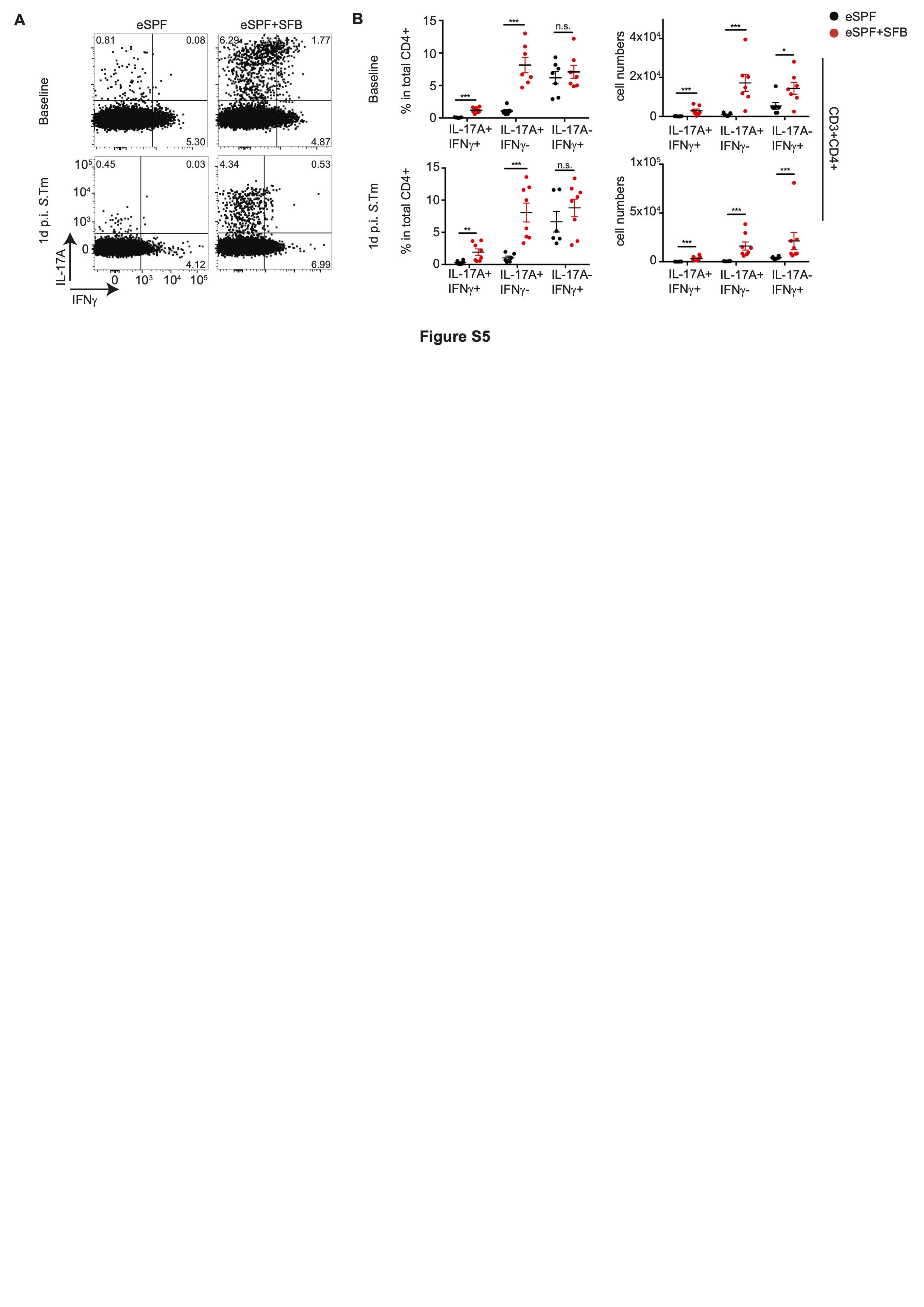

Supplement: Supplementary Figure S5 — IL-22 producing CD4 T cells are induced by SFB independent of IFNg+ CD4 T cells (A–B) LPL were isolated from baseline and 1d post S. Tm. infection from small intestine of IL-17AGFP IFN-γKatushka FoxP3RFP mice harboring eSPF or eSPF+SFB. Representative FACS plots (A) and percentages and absolute numbers (B) of CD3+CD4+ cells expressing IL-17A and IFN-γ without any ex vivo restimulation. Data represent n=6-9 mice/group as mean ± SEM from at least two independent experiments. P values indicated represent a unpaired Student’s t test *p < 0,05; **p < 0,01; ***p < 0,001; ****p< 0,0001. [file Image_5.jpeg]

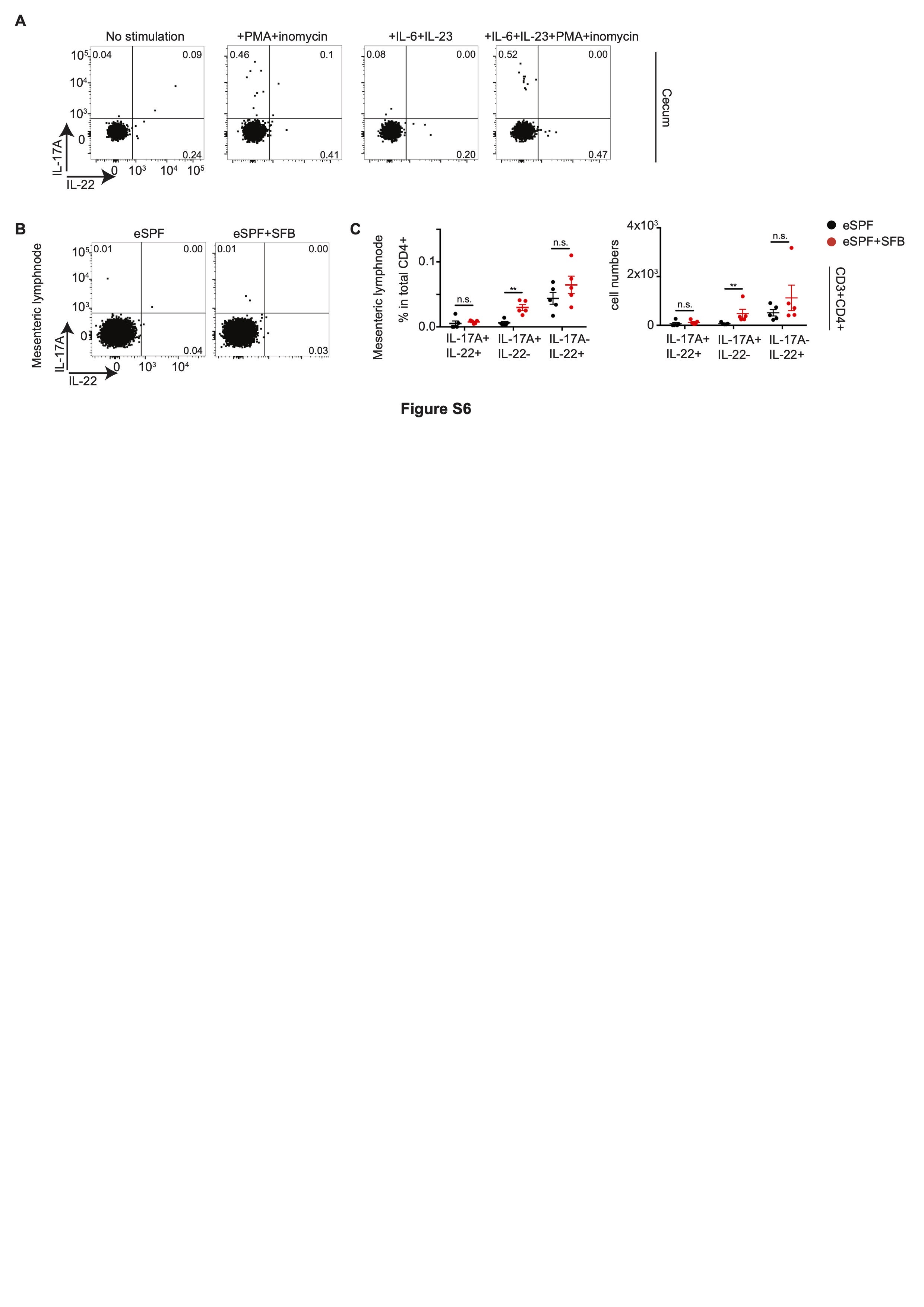

Supplement: Supplementary Figure S6 — SFB induced CD4 T cells during steady state require cytokine stimulation to secret IL-22 (A) Cecal LPL were isolated from baseline of IL-17AGFP IL-22BFP FoxP3RFP mice harboring eSPF+SFB. Isolated LPL were stimulated for 5hrs upon indicated conditions and analyzed by FACS. (B–C) Leukocytes were isolated from mesenteric lymphnode from IL-17AGFP IL-22BFP FoxP3RFP mice harboring eSPF or eSPF+SFB. Isolated LPL were stimulated for 5hrs in presence of IL-6, IL-23, PMA and inomycin and analyzed by FACS. Left, representative FACS plots (B) and right, frequencies (C) of CD3+CD4+ cells expressing IL-17A and IL-22. Data represent n=4-5 mice/group as mean ± SEM from at least two independent experiments. P values indicated represent a unpaired Student’s t test *p < 0,05; **p < 0,01; ***p< 0,001; ****p < 0,0001. [file Image_6.jpeg]

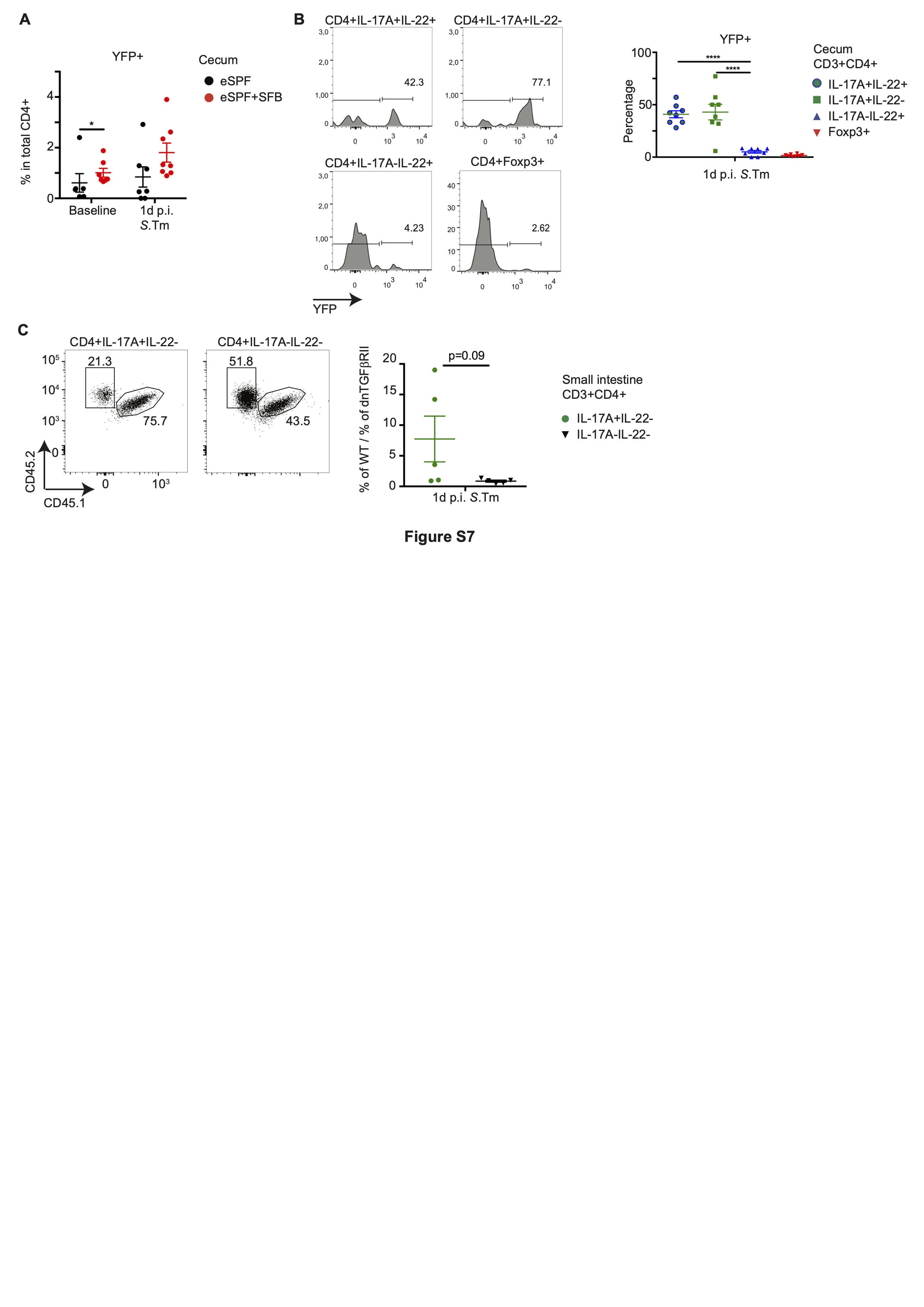

Supplement: Supplementary Figure S7 — SFB induced Th-22 cells development is independent of IL-17 secretion (A–B) CD3+CD4+ T cells from cecal LPL from eSPF and eSPF+SFB colonized Fate reporter mice were isolated 1d post S. Tm infection and analyzed for YFP expression during baseline. YFP expression in total CD3+CD4+ cells (A). YFP expression in IL-17A+ and/or IL-22+ cells and Foxp3+ cells from eSPF+SFB colonized mice (B) and in left, representative FACS plots and right, frequencies of YFP+ cells in CD4 T cells expressing IL-17A and/or IL-22 and Foxp3 without any ex vivo restimulation. (C) CD3+CD4+ T cells from small intestinal LPL after 1d S. Tm. infection from eSPF+SFB colonized Rag2-/- mice, that received CD4+ T cells from congenically labeled WT and CD4dnTGFβRII mice (1 : 1), were analyzed for expression of congenic markers in IL-17A+ and Foxp3+ cells. Left, representative FACS plots and right, frequencies of YFP+ cells in CD4 T cells expressing IL-17A and/or IL-22 and Foxp3 without any ex vivo restimulation. Data represent n=5-8 mice/group as mean ± SEM from at least two independent experiments. P values indicated represent an unpaired Student’s t test *p < 0,05; **p < 0,01; ***p< 0,001; ****p< 0,0001. [file Image_7.jpeg]
